# Improving five-year survival prediction via multitask learning across HPV-related cancers

**DOI:** 10.1371/journal.pone.0241225

**Published:** 2020-11-16

**Authors:** Andre Goncalves, Braden Soper, Mari Nygård, Jan F. Nygård, Priyadip Ray, David Widemann, Ana Paula Sales

**Affiliations:** 1 Lawrence Livermore National Laboratory, Livermore, CA, United States of America; 2 Cancer Registry of Norway, Oslo, Norway; University of Wisconsin, UNITED STATES

## Abstract

Oncology is a highly siloed field of research in which sub-disciplinary specialization has limited the amount of information shared between researchers of distinct cancer types. This can be attributed to legitimate differences in the physiology and carcinogenesis of cancers affecting distinct anatomical sites. However, underlying processes that are shared across seemingly disparate cancers probably affect prognosis. The objective of the current study is to investigate whether multitask learning improves 5-year survival cancer patient survival prediction by leveraging information across anatomically distinct HPV related cancers. Data were obtained from the Surveillance, Epidemiology, and End Results (SEER) program database. The study cohort consisted of 29,768 primary cancer cases diagnosed in the United States between 2004 and 2015. Ten different cancer diagnoses were selected, all with a known association with HPV risk. In the analysis, the cancer diagnoses were categorized into three distinct topography groups of varying specificity. The most specific topography grouping consisted of 10 original cancer diagnoses differentiated by the first two digits of the ICD-O-3 topography code. The second topography grouping consisted of cancer diagnoses categorized into six distinct organ groups. Finally, the third topography grouping consisted of just two groups, head-neck cancers and ano-genital cancers. The tasks were to predict 5-year survival for patients within the different topography groups using 14 predictive features which were selected among descriptive variables available in the SEER database. The information from the predictive features was shared between tasks in three different ways, resulting in three distinct predictive models: 1) Information was not shared between patients assigned to different tasks (single task learning); 2) Information was shared between all patients, regardless of task (pooled model); 3) Only relevant information was shared between patients grouped to different tasks (multitask learning). Prediction performance was evaluated with Brier scores. All three models were evaluated against one another on each of the three distinct topography-defined tasks. The results showed that multitask classifiers achieved relative improvement for the majority of the scenarios studied compared to single task learning and pooled baseline methods. In this study, we have demonstrated that sharing information among anatomically distinct cancer types can lead to improved predictive survival models.

## Introduction

Humans have the ability to transfer relevant knowledge from previous experiences to new ones, mastering them more easily and faster. Analogously in machine learning, multitask learning (MTL) methods attempt to improve model generalization and performance by using shared representations to exploit commonalities and differences across related tasks, while avoiding using information from unrelated tasks [1, 2]. MTL has shown to be an effective approach for overcoming the challenges of low quality datasets, such as scarce or highly skewed training data, that can degrade predictive performance [3–6].

While MTL has a history of over 20 years in machine learning, only recently has it started making its way into the field of predictive oncology. In the vast majority of such applications, MTL methods (and the related methods of transfer learning) are used to allow sharing of information across related datasets of the same type of cancer (e.g., breast cancers [7], skin cancers [8], and lung or prostate cancers [9]). In other words, tasks are defined in terms of datasets limited to a specific type of cancer.

However, as stark as the differences between distinct cancers may be, there probably exist underlying processes that are shared, even across seemingly very disparate cancer types. For example, it is known that human papillomavirus (HPV) plays a role in roughly 5% of all cancers worldwide [10]. While HPV is considered a necessary cause of cervical cancer, it is also linked to cancers in other anatomical sites, with rates varying according to susceptibility to oncogenic types of HPV. For instance, 90% of anal and 74% of vaginal cancers appear to be induced by HPV [11, 12]. Nearly 30% of penile and vulvar cancers may be caused by this virus [13, 14]. Likewise, about 30% of all head and neck cancers are HPV positive [15]. This proportion has been increasing over time, approaching 80% of incident tonsil cancers in some countries [16]. In addition to the similarities in the etiology, better survival has been observed among patients with HPV-related cancers who were tested positive for HPV, such as patients with penile cancer [17, 18], nasopharyngeal cancer [19], anal cancer [20], and vulvar cancer [17, 21]. In contrast to traditional tobacco- and alcohol-associated oropharyngel cancers, patients with positive HPV findings have demonstrated improved survival and significantly higher cure rates [22–24]. In spite of the commonalities described for HPV-related cancers, these patients have been managed through different oncology disciplines such as gynecologic oncologist, otolaryngologist or head and neck surgeons, onco-gastroenterologists, and onco-urologists. With low annual incidence rate, some less than 4 per 100,00 individuals, the clinical experience is not only modest, but also rarely shared across the segregated fields of oncology.

In the majority of developed countries, oncology care units regularly report selected information on each cancer patient to the nation-wide cancer registry surveillance program. While the information sent to the surveillance programs is less nuanced than information available in the clinics, it is standardized following international conventions and the quality of the selected variables are formally assured for accuracy over time. Data-driven statistics are computed on incidence, mortality and survival, informing aspects on evidence-based care and changes in disease burden on a regular basis. Consequently, cancer registry data has been a valuable source of research for hypothesis generation and testing [25–27].

Encouraged by recent developments in the field of multitask learning (MTL) and increased availability of accurate registry data, our aim is to investigate whether MTL can be used to leverage registry data for 5-year survival prediction. Potentially, the prediction performance for rare cancer types with limited patient records may be improved via leveraging patient records of similar cancer types but with larger number of patient records. Hence, as opposed to sharing information across different datasets of the same cancer type, we approach MTL for cancer from a different perspective: leverage MTL to share information across anatomically distinct cancer types. In this context, HPV related cancers serve an excellent model to test the accuracy of the different survival prediction models.

Many existing MTL methods rely on explicit assumptions about the relationships between tasks. These assumptions are incorporated into machine learning algorithms through specifically designed priors [6, 28] or regularization functions [29–31]. More recently proposed methods are capable of learning the relationships between tasks from the data and incorporating this information into the learning process [4, 5]. The MTL method proposed in this study is built upon the MSSL approach proposed in Goncalves et al. [5], which, aside from learning tasks coefficients, also estimates the relationship among the tasks represented as an undirected graph. This is useful in inferring how information is shared across the different tasks during model training.

When predicting whether or not patient will survive for at least five years (i.e., binary classification problem) based on patient features, it is important that the learning algorithms are capable of handling censored data, to avoid potential bias [32]. In Vock et al. [33], a general-purpose technique for adapting machine learning algorithms to right-censored, time-to-event data is presented. The method is based on computing inverse probability of censoring weights (IPCW) which are then used to construct a weighted loss function and weighted performance metrics used in the training and testing of the given learning algorithm. In this paper, we extend the MSSL formulation of Goncalves et al. [5] to appropriately handle right-censored data using the inverse probabilities of censoring weights.

In summary, the objective of the current study is to investigate whether multitask learning improves 5-year survival cancer patient survival prediction by leveraging information across anatomically distinct HPV related cancers.

## Material and methods

### Cohort selection

Data was obtained from the Surveillance, Epidemiology, and End Results (SEER) program database [34], which provides de-identified information on cancer statistics of the United States’ population. Specifically, data from nine SEER registries were used: Atlanta, Connecticut, Detroit, Hawaii, Iowa, New Mexico, San Francisco-Oakland, Seattle-Puget Sound, and Utah. Although data is available for cases diagnosed from 1973 through 2015, we only used data from 2004 onwards due to the fact that in that year there was a major change in the criteria used for both cancer stage and grade definitions. In this study, we focus on cancers in anatomical sites for which evidence of an association between HPV and cancer has been established. Based on evidence suggesting etiological link between infection with human papillomavirus (HPV) infection and cancer, we selected the following cancer sites to the study using the International Classification of Diseases for Oncology, 3rd edition (ICD-O-3) topography codes [35]: cervix (C53), anus and anal canal (C21), vulva (C51), vagina (C52), and penile cancer (C60). Regarding head and neck cancers, we included all cancers coded as C01, C09, and C10 where all sub-sites are HPV related [36]. C02, C05 and C11 are types of head and neck sited with mixed etiology in respect to HPV infection. Regarding cancers coded as C02 (other and unspecified parts of tongue), sub-sites C02.0-3 and C02.9 have been referred to as not HPV-related and therefore not included, while sub-sites C02.4 and C02.8 are linked to HPV infection and are included in this study. Regarding cancers coded as C05 (palate) sub-sites C05.1 (soft palate) and C05.2 (uvula) are HPV related (included) while sub-sites C05.8 and C05.9 are typically not HPV related (excluded). We did not include cancers in lip, gum, floor and other unspecified parts of mouth, cancers in glands, sinuses and hypopharynx due to the lack of strong evidence of being associated with HPV-infection. We also excluded C11 (nasopharynx), which is linked to infection with Ebstein-Barr virus [37] and where the HPV-etiology is not firmly established [38] partly because this site is difficult to study as the deep structures of the skull base related to the nasopharynx are inaccessible to routine clinical examinations.

In addition we removed all cases for which: 1) survival time and/or event information were missing; 2) age at diagnosis was under 18 years; 3) the case is a pre-cancer (cases in which the cancer stage is 0); 4) the number of survival months after being diagnosed is zero; 5) the cancer was not the first diagnosed cancer case of the patient.

After exclusions, the cohort consisted of 29,768 primary cancer cases diagnosed in the United States between 2004 and 2015 with anatomical sites associated with HPV risk. The cohort contains a total of 11,887 men and 17,818 women, with mean age of 57 years. A descriptive table of the population data used in this paper is shown in Table 1.

**Table 1:** Population data description per anatomical site, classified by ICD-O-3 topography codes: Number of cancer cases (N), age information, and 5-year survival rate.

ICD-O-3 code	Anatomical site	N	Age			5 year Survival (%)
			mean	median	min–max	
C01	Base of tongue	4421	61.03	60	19–102	63.3
C02	Other/Unsp tonge *	244	59.79	59	26–96	59.7
C05	Palate**	488	61.06	61	20–101	56.2
C09	Tonsil	5511	57.91	57	19–102	72.0
C10	Oropharynx	943	60.79	59	25–94	42.2
C21	Anus & Anal canal	4287	60.29	59	19–105	63.5
C51	Vulva	2733	66.11	66	19–102	62.6
C52	Vagina	645	65.43	64	23–100	41.9
C53	Cervix Uteri	9729	48.90	47	19–103	68.7
C60	Penis	767	65.85	66	26–98	58.0
	Total	29,768	57.05	57	19–105	62.5

*Subsite C02.0-3/9 excluded.

**Subsite C05.8/9 excluded.

### Tasks definition

In MTL an important step is to define the “tasks”. In many applications of MTL, tasks are easily identified. In our application, defining the tasks is not straightforward, as there are many possible ways to do so. We next provide details of our criteria for tasks definition.

Because we aim to implement MTL methodologies across cancer types, the task definition will be determined by how cancer “type” is defined. For the purposes of this study we will define a cancer type based on the anatomical location of the cancer diagnosis. We consider three distinct strategies for grouping individual cancer sites with a decreasing degree of specificity. The most specific grouping, *Topography group 1* (TP1), consists of 10 cancer sites which are defined by the first two digits of the ICD Topography code. In the next most specific grouping, *Topography group 2* (TP2), cancers from *Topography group 1* were grouped by organ, resulting in a total of six cancer sites. Finally, in the least specific grouping, *Topography group 3* (TP3), related organs are grouped into broad anatomical regions, resulting in just two cancer sites. Table 2 presents the groupings utilized in our experiments.

**Table 2:** ICD-O-3 codes included in the cohort and topography group division used in our experiments.

	PRIMSITE	Topography Group 1		Topography Group 2		Topography Group 3	
		Task ID		Task ID		Task ID	
Tasks	C01	T-01:	Base Tongue	T-01:	Tongue	T-01:	Head & Neck
	C02	T-02:	Other/Unsp. Parts of Tongue				
	C05	T-03:	Palate	T-02:	Palate		
	C09	T-04:	Tonsil	T-03:	Oropharynx & Tonsils		
	C10	T-05:	Oropharynx				
	C21	T-06:	Anus & Anal Canal	T-04:	Anus & Anal Canal	T-02:	Ano-Genital
	C51	T-07:	Vulva	T-05:	Genital Female		
	C52	T-08:	Vagina				
	C53	T-09:	Cervix Uteri				
	C60	T-10:	Penis	T-06:	Genital Male		

Topography group 1 through 3 represent groupings of ICD-O-3 codes with decreasing degree of specificity.

Given a particular topography group, a task is defined as the binary classification problem of predicting whether a patient with a cancer diagnosis at a particular cancer cite (as defined by the topography group) will survive less than five years or more from the time of diagnosis. Demographic and cancer-related information are used as predictive features. By looking into distinct topography groups of anatomical specificity, we aim to investigate the performance of the multitask learning methods under the different strategies of splitting the data into tasks.

### Variable selection and re-coding

Inspired by the work of Lynch et al. [39], a total of 14 predictive variables, listed in Table 3, are chosen. Variables with prefix “X_” are derived from features in the SEER database. The process is described in the next paragraph. Variables indicated with an asterisk are re-coded versions of the original variables in the SEER database. The re-coding process for each of these variables are presented in the S1 File. The main reason for re-coding is to group similar categories into larger groups, reducing the number of categories for the modelling to be more effective, without loosing predictive power.

**Table 3:** Variables obtained from SEER database and used as 5-year survival prediction features.

SEER variable	Description	Type	# Levels
REG	Registry ID	Categorical	9 levels
AGE_DX	Age at diagnosis	Numerical	-
SEX	Sex	Categorical	2 levels
RAC_RECA	Race recode (White, Black, Other)	Categorical	5 levels
SURGSCOF	Scope of regional lymph node surgery	Categorical	3 levels
HISTREC	Histology recode, broad groupings	Categorical	3 levels
*GRADE	(Recoded) Grade	Numerical	-
*DAJCCT	(Recoded) AJCC ‘T’ component (6th Ed.)	Numerical	-
*DAJCCN	(Recoded) AJCC ‘N’ component (6th Ed.)	Numerical	-
*DAJCCM	(Recoded) AJCC ‘M’ component (6th Ed.)	Numerical	-
*DAJCCSTG	(Recoded) AJCC ‘stage group’ component (6th Ed.)	Numerical	-
*SURGPRIF	(Recoded) Surgery of primary site, generic	Numerical	-
X_PRIMSITE_1	First two digits of ICD-O-3 code for anatomical site	Categorical	10 levels
X_TUMSIZ_COMB_NUM	Tumor size	Numerical	-
SURV_TIME_MON	Survival time in months	Numerical	-
X_SURV_TIME_5Y	Five year survival	Binary	-

Variables indicated with “*” are re-coded versions of the original SEER variables (see S1 File). Variables with prefix “X_” refer to modified variables derived from original SEER variables (see text). All other variables are kept as originally coded. SURV_TIME_MON and X_SURV_TIME_5Y are predictands of the model, thus they are not considered in the input feature set. AJCC stands for American Joint Committee on Cancer.

X_PRIMSITE_1 is derived from SEER variable PRIMSITE, such that the first two digits of PRIMSITE are assigned to X_PRIMSITE_1. X_TUMSIZ_COMB_NUM is derived from SEER variable CSTUMSIZ, which contains both numeric values (actual sizes of tumors in millimeters) as well as ordinal values (codes that indicate that the tumor size lies within a range of 10 millimeters). In X_TUMSIZ_COMB_NUM all the numeric values are retained while the categorical values were mapped to the median of the range of the bin to which the given tumor was assigned.

Variables other than the ones with prefix “X_” or “*” are maintained as originally coded.

### Feature encoding

To be used by the machine learning algorithms, all predictive variables must be represented numerically. This process is referred to as *feature encoding*. Different encoding strategies are applied to different types of predictive variables, the selection of which depends on the characteristics of the variable under consideration. In what follows we use the terms variable and feature interchangeably.

Numerical predictive variables such as AGE_DX and X_TUMSIZ_COM_NUM are already real numbers, so they can directly be used as features by the methods without any additional encoding.

Variables that are purely categorical (qualitative variables with no clear ordering or associated numerical values) are usually represented via one-hot-encoding. In this encoding strategy the variable is represented by a binary vector of the same size as the number of categories. The vector contains zeros everywhere except for the position corresponding to the given category, in which case a 1 appears. This is equivalent to introducing dummy variables for categorical variables in standard regression analysis. The following variables are encoded using this strategy: REG, SEX, RAC_RECA, HISTREC, X_PRIMSITE_1, SURGSCOF, and X_SURGPRIF_GEN. Cases with “Unknown” / “Not Applicable” categories are treated as an additional category for that feature.

Stage-related variables have an intrinsic ordering of the categories. For example, the categories in the feature DAJCCSTG have an increasing order related to the severity of the cancer diagnosis: stage I, stage II, stage III, and stage IV. To preserve this ordinal relationship we used a label encoding strategy in which each stage category is assigned an integer value corresponding to its relative severity. For example, in the feature DAJCCSTG stage I is represented by ‘1’, stage II by ‘2’ and so on. However, in the SEER dataset these variables also have an “Unknown” / “Not applicable” category that breaks the natural ordering of the categories. To deal with these cases, we propose to represent this particular category as the *empirical mean* of all assigned integer valued labels in the observed data. Note that this process is a type of imputation which treats the “Unknown” / “Not applicable” category as data that is missing at random. We used this encoding approach for the following features: GRADE, DAJCCT, DAJCCN, DAJCCM, and DAJCCSTG.

### Outcome definition

A binary outcome, X_SURV_TIME_5Y, derived from the SEER feature SRV_TIME_MON, was used as the outcome variable. A value of 1 indicates that a patient has survived at least five years from the time of diagnosis, and a value of 0 indicates that the patient survived less than five years from the time of diagnosis. Five-year survival has been the de facto method for reporting cancer survival in major epidemiological studies, the most recent being the Concorde Programme [40]. The use of five-year survival originates from the fact that until recently, cancer was a fatal disease and patients surviving for that long could be considered cured [41]. Although the proportion of patients who survive for 5 years has been increasing over the years, it remains a widely used benchmark, even though it cannot be directly interpreted as the proportion of patients who are cured [42].

### Censored cases

The censor variable used in this study was built from SEER’s variable STAT_REC, which describes whether the patient is dead or alive at the end of follow-up. All follow-up is censored at the cut-off date (Dec 31st, 2015). Any patient that dies after this date is considered alive as of the cut-off date. Since we are focusing on 5-year survival prediction, any patient that is alive but its survival time (SRV_TIME_MON) is less than 60 months, due to the cut-off date, is considered censored. Therefore, all alive cases that were diagnosed after 2011 are censored. If STAT_REC indicates death, then it is uncensored. In case the patient is alive and has already survived for at least 60 months, then it is uncensored.

## Methods

To evaluate our hypothesis that combining data from apparently disparate cancer types could lead to model performance improvements, a multitask classifier was compared against two single task baselines. The three methods are described below in the section *Classifiers*.

Our learning tasks are classification problems using distinct datasets. We denote by *T* the number of tasks, *d* the number of features in each dataset, assumed to be identical for all learning tasks, and *n*_*t*_ the number of samples for the *t*-th task. $X_{t}\in R^{n_{t}\times d}$ and $y_{t}\in {\left\{0,1\right\}}^{n_{t}}$ are the feature (covariate) matrix and the binary outcome vector for the *t*-th task. $W\in R^{d\times T}$ is the MTL parameter matrix, where columns are vector parameters $w_{t}\in R^{d}$, *t* = 1, …, *T*, for each task. For any matrix **A**, tr(**A**) is the trace operator and ‖**A**‖_1_ is the *ℓ*_1_-norm of matrix **A**, defined as the sum of the absolute values of its entries.

### Treatment of censored data

We applied a general-purpose technique for adapting machine learning algorithms to right-censored time-to-event data [33]. Inverse probability weighting is a method of constructing estimators and likelihood functions that account for sampling biases and missing data [43]. The idea is to use the probability of being sampled, or estimates thereof, to calculate weights that adjust the number of unlikely samples by inflating their representation in the observed data to better reflect the true sampling population. For example, suppose the probability of being sampled is known, and a particular sample *x*_*i*_ has a probability of being sampled *p*_*i*_ > 0. The value $\frac{1}{p_{i}}$ can be used to weight the observed sample *x*_*i*_ when constructing estimators or likelihood functions to essentially create a set of pseudo-samples that increases the effective sample size of the observed data. Intuitively if *p*_*i*_ is close to one, then the sample was very likely to have been selected, thus no adjustment is needed. Consequently, $\frac{1}{p_{i}}$ is also close to one resulting in minimal adjustments. On the other hand if *p*_*i*_ is very small, then there are many more samples similar to *x*_*i*_ in the true population that were not selected. To represent these samples, the inverse probability weight $\frac{1}{p_{i}}$, which is now much larger than one, is used to inflate the number of samples similar to *x*_*i*_ in the observed data. More concretely, if $p_{i}=\frac{1}{10}$ then on average for every *x*_*i*_ in the observed data, there are 10 such samples in the true population that were not chosen. Thus we inflate the number of samples of the type *x*_*i*_ by a factor of $\frac{1}{p_{i}}=10$.

Censoring is a type of sampling bias, and the method of inverse probability of censoring weights (IPCW) constructs weights that use the empirical distribution of censoring times to compute inverse probability weights to adjust estimators and likelihood functions in survival models. To see how censoring can lead to biased predictions in the case of binary classification, consider the following standard survival analysis set-up. Indexing patients by *i*, let *t*_*i*_ be the survival time, *c*_*i*_ the censoring time and **x**_*i*_ the features of patient *i*. We assume the existence of a joint probability distribution *P*(*c*, *t*, **x**) such that the *complete data* is (*c*_*i*_, *t*_*i*_, **x**_*i*_) ∼ *P*(*c*, *t*, **x**). Defining *v*_*i*_ = min{*c*_*i*_, *t*_*i*_} and *δ*_*i*_ = *I*(*t*_*i*_ < *c*_*i*_), where *I*(⋅) is the indicator function, the *observed data* is (*v*_*i*_, *δ*_*i*_, **x**_*i*_). Traditional survival analysis seeks to make inference about the distribution *P*(*t*|**x**) given the observed data {*v*_*i*_, *δ*_*i*_, **x**_*i*_}. In the binary classification problem, we want to predict whether patient *i* survives for *τ* > 0 years after the date of diagnosis, given the data **x**_*i*_. Define the binary random variable *y*_*i*_ = *I*(*t*_*i*_ ≥ *τ*) with expected value *π*(**x**_*i*_) = *E*(*y*_*i*_|**x**_*i*_). In the presence of right-censoring, the value of *y*_*i*_ will be unknown for some patients, namely those patients with *v*_*i*_ < *τ* and *δ*_*i*_ = 0. Restricting analysis to patients with known *y*_*i*_ will lead to bias in the predictions since patients with small event times, i.e. small *t*_*i*_, are less likely to be censored. Thus we will over sample patients with *y*_*i*_ = 0, leading to potentially biased predictions.

To correct for this oversampling, IPCW can be used to essentially artificially inflate the dataset by weighting the influence of uncensored individuals with large event times. The intuition behind this weighting is that the longer a patient survives, the more unobserved patients with similar survival times there are that dropped out due to censoring. These longer-surviving patients are weighted to represent these unobserved censored patients. Weights are computed via inverse probabilities [43] and require estimates of the censoring distribution’s survival function (the complement of the cumulative distribution function). If we assume that the censoring distribution is independent of event times and patient features, i.e. *P*(*c*, *t*, **x**) = *P*(*c*)*P*(*t*, **x**), then we can estimate the censoring distribution’s survival function via the Kaplan-Meier estimator, which is defined as

$$\begin{array}{c} \hat{G}\left(t\right)=\prod_{i:v_{i}<t}(1-\frac{k_{i}}{m_{i}}), \end{array}$$ (1)

where *k*_*i*_ are the number of events observed at time *v*_*i*_ and *m*_*i*_ is the number of individuals who have not yet experienced an event and are not yet censored just before time *v*_*i*_. The IPCW for patient *i* is then defined as

$$\begin{array}{c} \omega_{i}=\{\begin{array}{cc} \frac{\delta_{i}}{\hat{G}\left(\text{min}\left\{v_{i},\tau \right\}\right)} & \text{if}\;\text{min}\left\{t_{i},\tau \right\}<c_{i},\\ 0 & \text{otherwise.}\; \end{array} \end{array}$$ (2)

Note that patients who are censored before the threshold *τ* have a weight of zero and do not contribute directly to the data, but instead are incorporated indirectly through the weights. As outlined in [33], these weights can be used to adjust loss functions and performance metrics to account for right-censored data.

### Classifiers

In this study, in addition to the MTL classifier, two baseline methods were considered: Single task learning (STL) models and pooled models. These two baselines represent the two extremes in the spectrum of information sharing across cancer types. At one end of the spectrum, STL utilizes one model per task, so there is no sharing of any information across tasks. At the other end, the pooled model utilizes a single model for all tasks, so all information is shared across all tasks. The MTL method lies somewhere in between these two baselines, providing a principled way of controlling the level and nature of information sharing across subsets of tasks.

#### Baseline 1: Single task classifiers

This baseline consists of building individual classifiers separately for each task, i.e., cancer type (listed in Table 2). The data presented to each classifier consists of samples from the same cancer group, and hence is much more homogeneous than the data presented to the other two types of classifiers described below. However, as some cancer groups have low incidence, the corresponding STL classifiers are trained with relatively small training sets. The classification model is a *ℓ*_1_-penalized (lasso) logistic regression. The choice for *ℓ*_1_-penalization is due to its variable selection property and success in practical applications [44, 45].

To deal with censored data inputs, we adapt the lasso logistic regression formulation using the method proposed in Vock et al. [33] discussed in section *Treatment of censored data*. The result is a weighted version of the traditional *ℓ*_1_-penalized logistic regression. The adapted formulation is defined as

$$\begin{array}{c} w=\underset{w}{\text{arg}\;\text{min}}\;\frac{1}{n}\sum_{i=1}^{n}\omega_{i}L\left(y_{i},x_{i},w\right)+\lambda {\left\|w\right\|}_{1}, \end{array}$$ (3)

where $L(y_{i},x_{i},w)$ is the cross-entropy loss function, ***ω*** the IPCW weights, *y*_*i*_ ∈ {0, 1} and $x_{i}\in R^{d}$ are the label and features of the *i*-th data instance, and λ ≥ 0 is a hyper-parameter that controls the amount of regularization. The cross-entropy loss function is defined as:

$$\begin{array}{c} L\left(y_{i},x_{i},w\right)=-\left(y_{i}\;\text{log}\left(\sigma \left(w^{⊺}x_{i}\right)\right)+\left(1-y_{i}\right)\;\text{log}\left(1-\sigma \left(w^{⊺}x_{i}\right)\right)\right) \end{array}$$

where *σ*(⋅) is a sigmoid function. Note that in Eq 3, λ must be specified by the user or selected via cross-validation. The regularization aims to attenuate the overfitting that is likely to occur, particularly for tasks with small sample sizes. Our implementation of the IPCW *ℓ*_1_-penalized logistic regression is based on the Scikit-learn package [46].

#### Baseline 2: Pooled classifier

The pooled baseline consists of a single classifier for the entire cohort. In this case, data from all tasks are pooled into one monolithic task and a single classifier is trained over all cancer types. The feature used to define the tasks in the MTL model (described in section *Classifiers*) and to define individual classifiers in Baseline 1 (described in section *Classifiers*), is passed to this classifier as an additional predictor feature. Similar to the model in Baseline 1, our implementation of the IPCW *ℓ*_1_-penalized logistic regression uses Scikit-learn [46]. The advantages of this pooled classifier baseline are that the training set is much larger (albeit more heterogeneous compared to Baseline 1), and smaller model complexity than MTL, which implies a lower risk of model overfitting with smaller datasets. This pooled classifier makes the strong assumption that all tasks have a high level of similarity, ignoring particularities of individual cancer groups.

#### Multitask learning classifier

For this study, we extended the MTL formulation proposed in Goncalves et al. [5] called Multi-task Sparse Structure Learning (MSSL) to deal with right-censored data using the IPCW adaptation. The MSSL formulation has shown promising results on classification problems from a variety of domains. Aside from learning task coefficients, MSSL also estimates the relationship among the tasks represented by an undirected graph, which can be further analyzed. The adapted IPCW-MSSL formulation is defined as

$$\begin{array}{c} W=\underset{W,\Omega ⪰0}{\text{arg}\;\text{min}}\sum_{t=1}^{T}\frac{1}{n_{t}}\sum_{i=1}^{n_{t}}\omega_{i}^{t}L\left(y_{i}^{t},x_{i}^{t},w_{t}\right)+\lambda_{1}\text{tr}\left(W\Omega W^{T}\right)-d\;\text{log}|\Omega |+\lambda_{2}{\left\|\Omega \right\|}_{1}, \end{array}$$ (4)

where $L(y_{t},X_{t},w_{t})$ is the cross-entropy loss function on the *t*-th task, and *d* is the problem dimension. The matrix **Ω** is an inverse covariance (precision) matrix that captures the dependence among tasks and is learned together with the task-specific parameters **W**. Finally, λ_1_ ≥ 0 and λ_2_ ≥ 0 are hyper-parameters that control the trade-off between the corresponding terms and need to be specified by the user. A detailed discussion on the role of each term is provided in Goncalves et al. [5]. For our experiments, we adapted the python code made publicly available by the authors.

### Experimental setup

For each experiment, we randomly selected 70% of the available data for training and the remaining 30% for testing. Each experiment was repeated 30 times with different random train/test partitions to account for the variability of training and test splits. In every repetition, the three methods received exactly the same training and test sets. The hyper-parameters of the methods were selected by cross-validation. The hyper-parameters values resulting in the smallest average performance metric (Brier score) over all tasks were selected.

To assess the performance of the methods, the Brier score [47] was used. In the MTL setting, there are two complementary approaches for evaluating predictive performance: 1) ‘per-sample basis’, where for each experiment repetition, test sets from all tasks are pooled together and a score is computed; and 2) ‘per-task basis’, where the performance metric is computed for each task individually, such that each task contributes equally regardless of sample size.

To determine whether the improvement in performance obtained by MTL was practically significant, two measures of effect size were used: *Cohen’s d* [48] and *common language effect size (CLES)* [49]. The notion of effect size is typically associated with randomized experiments with both control and treatment group. In our context, the population of Brier scores from randomly sampled test/train splits from the various algorithms constitute our control and treatment groups. Results from STL and Pooled models will be considered control groups while the MTL results will be the treatment group. Thus larger effect sizes are indicative of more significant differences in model performance. Each Brier score in the control group is paired with one of the treatment group by nature of the fact that they were trained on the same test/train split. Let $b_{i}^{c}$ and $b_{i}^{t}$ be the Brier scores from the *i*-th test/train split in the control group and the treatment group respectively. Let $\Delta_{i}=b_{i}^{c}-b_{i}^{t}$ be the difference of the *i*th Brier scores. Then Cohen’s *d* for paired samples is the sample mean of Δ_*i*_ divided by the sample standard deviation of the Δ_*i*_:

$$\begin{array}{c} d=\frac{{\bar{\Delta }}_{i}}{SD(\Delta_{i})}. \end{array}$$

As an empirical measure of the signal-to-noise ratio, larger values are indicative of a larger effect of the treatment (MTL). In contrast to Cohen’s *d*, CLES is a probabilistic measure of effect size. Specifically, CLES is defined as the probability that two individuals randomly chosen from each population will differ in a particular way. In our case, we would like to know if the mean Brier score is significantly less in the treatment group than the control group. Thus in our case CLES is the probability that a randomly chosen Brier score from the treatment group is less than a randomly chosen Brier score from the control group. Because our samples are paired we simply compute the fraction of paired samples in which the treatment group’s Brier score is lower than the control group’s:

$$\begin{array}{c} CLES=\frac{1}{n}\sum_{i=1}^{n}1_{\left\{b_{i}^{c}>b_{i}^{t}\right\}}. \end{array}$$

All measures of effect size can be found in Appendix B of S1 Appendix.

## Results

The sample sizes for the different tasks vary widely, particularly for the most fine-grained task definition (*Topography group 1*). In this case, the total number of samples per task is as low as 244 for *other/unsp. parts of tongue* cancer (ICD code C02), to higher than 9,729 for *cervical* cancer (ICD code 53) (Table 1). Likewise, 5-year survival also varies significantly across tasks, with rates as low as 42.2% for *oropharyngeal* cancer (ICD code 10) and as high as 72% for *tonsil* cancer (ICD code C09).

**Fig 1:**
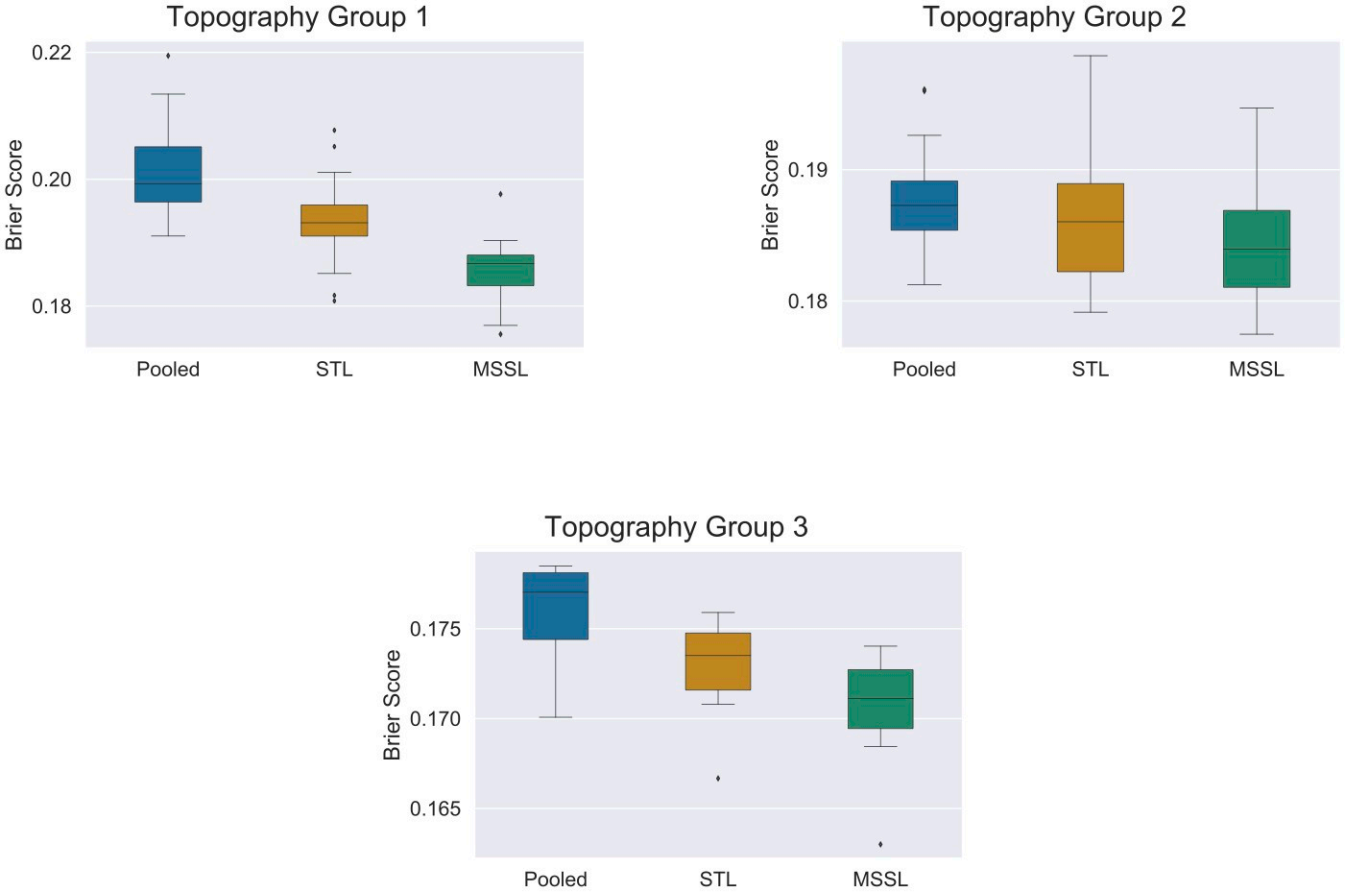
Brier score (y-axis) performance by classifiers (pooled, STL, and MSSL). Results show the aggregated performance from all tasks. Boxplots are composed of the mean Brier score over all 30 independent runs. MSSL shows superior performance in all three tasks splitting approaches.

We start by comparing MSSL, STL, and pooled classifiers on a per-sample basis for the three tasks split definitions. Brier score is used as the performance metric: lower Brier score indicates that the method has a better prediction performance. For each method we combined the true and predicted values from all tasks, and computed a single Brier score by combining all test observations, regardless of task assignment. The per-sample basis comparison was performed for each task definition. As shown in Fig 1, the MSSL classifier consistently outperformed both STL and pooled classifiers, across all task definitions. Specifically, we grouped the ICD-O-3 codes into two broader anatomical classes, listed in Table 2, and performed the same type of comparisons above. The groups were designed in increasing level of specificity within a hierarchy: *Topography group 1* (first two digits of ICD-O-3 anatomical codes) with 10 tasks, *Topography group 2* with 6 tasks, and finally *Topography group 3* with only 2 tasks. All models show a decreasing trend in the Brier score as we go from *Topography group 1* to *Topography group 3*. The best performance for all methods was obtained in the coarsest task split (*Topography group 3*). This is intuitively pleasing because in the coarsest task split we have more data per task, and all models benefit from this. Nevertheless, across all comparisons, the MSSL classifier outperformed both STL and pooled classifiers for the large majority of cases.

**Fig 2:**
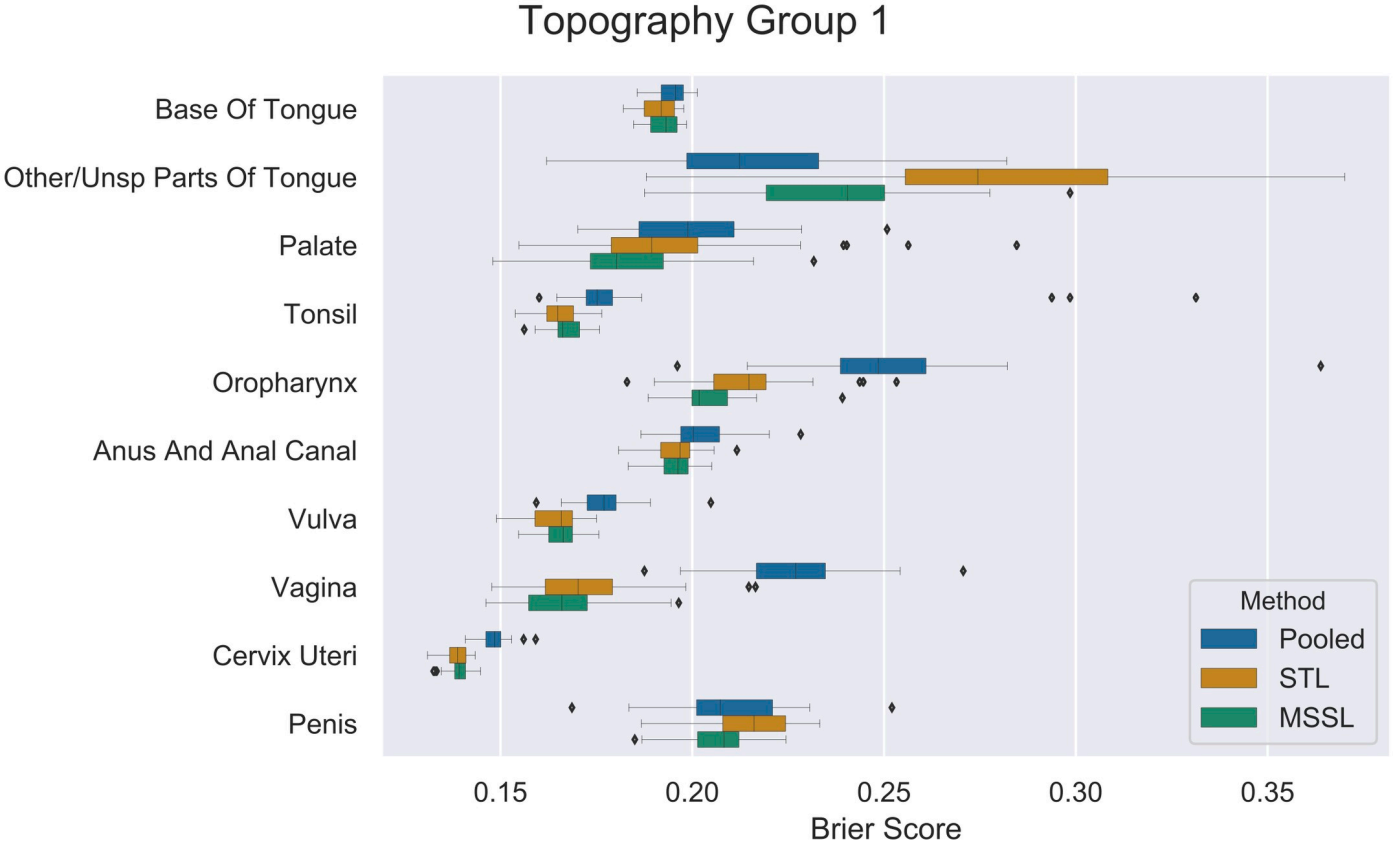
Brier scores (x-axis) for pooled, STL, and MSSL classifiers for *Topography group 1* data split. Boxplots show results from 30 independent runs.

**Fig 3:**
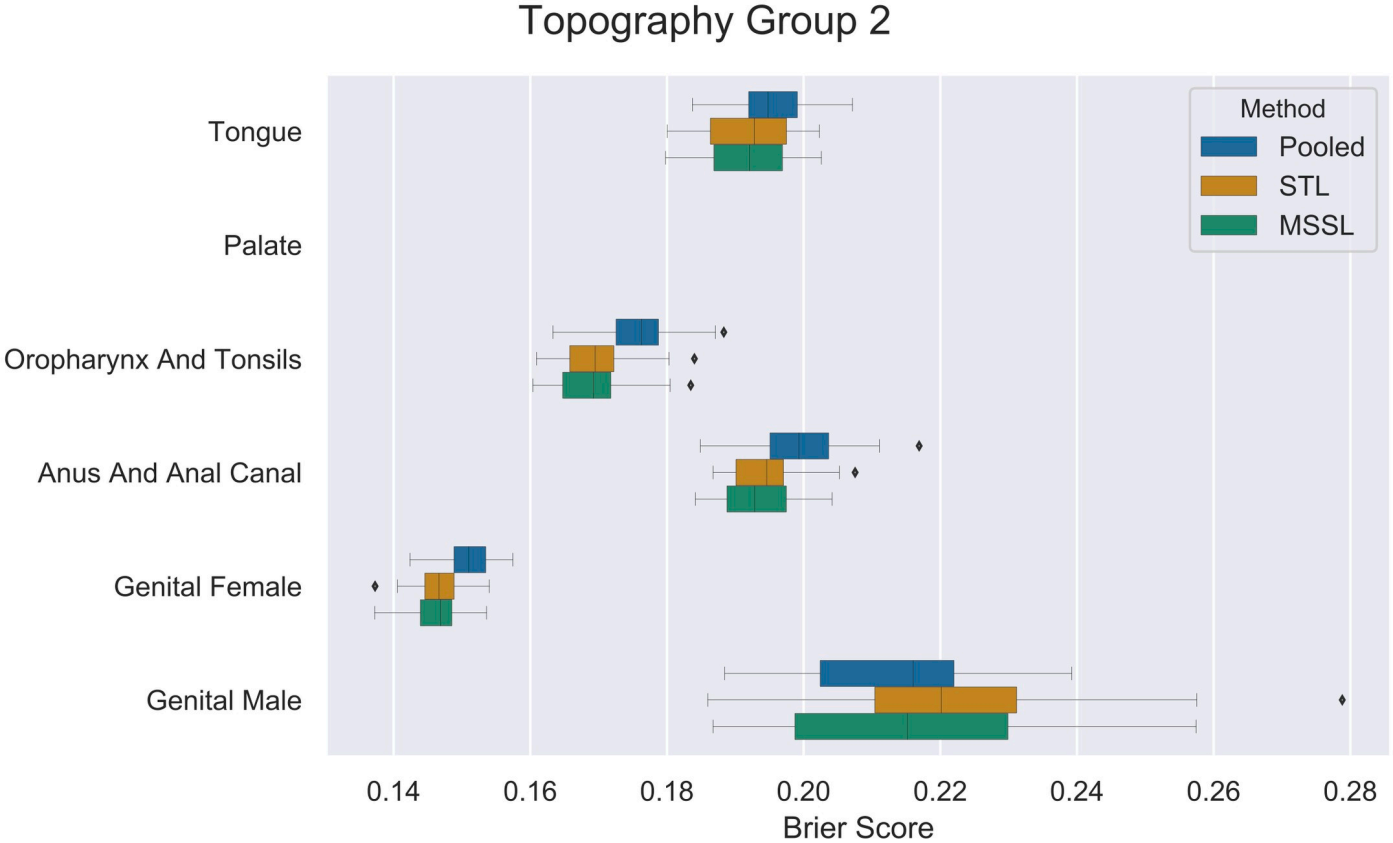
Brier scores (x-axis) for pooled, STL, and MSSL, and pooled classifiers for *Topography group 2* data split. Boxplots show results from 30 independent runs.

**Fig 4:**
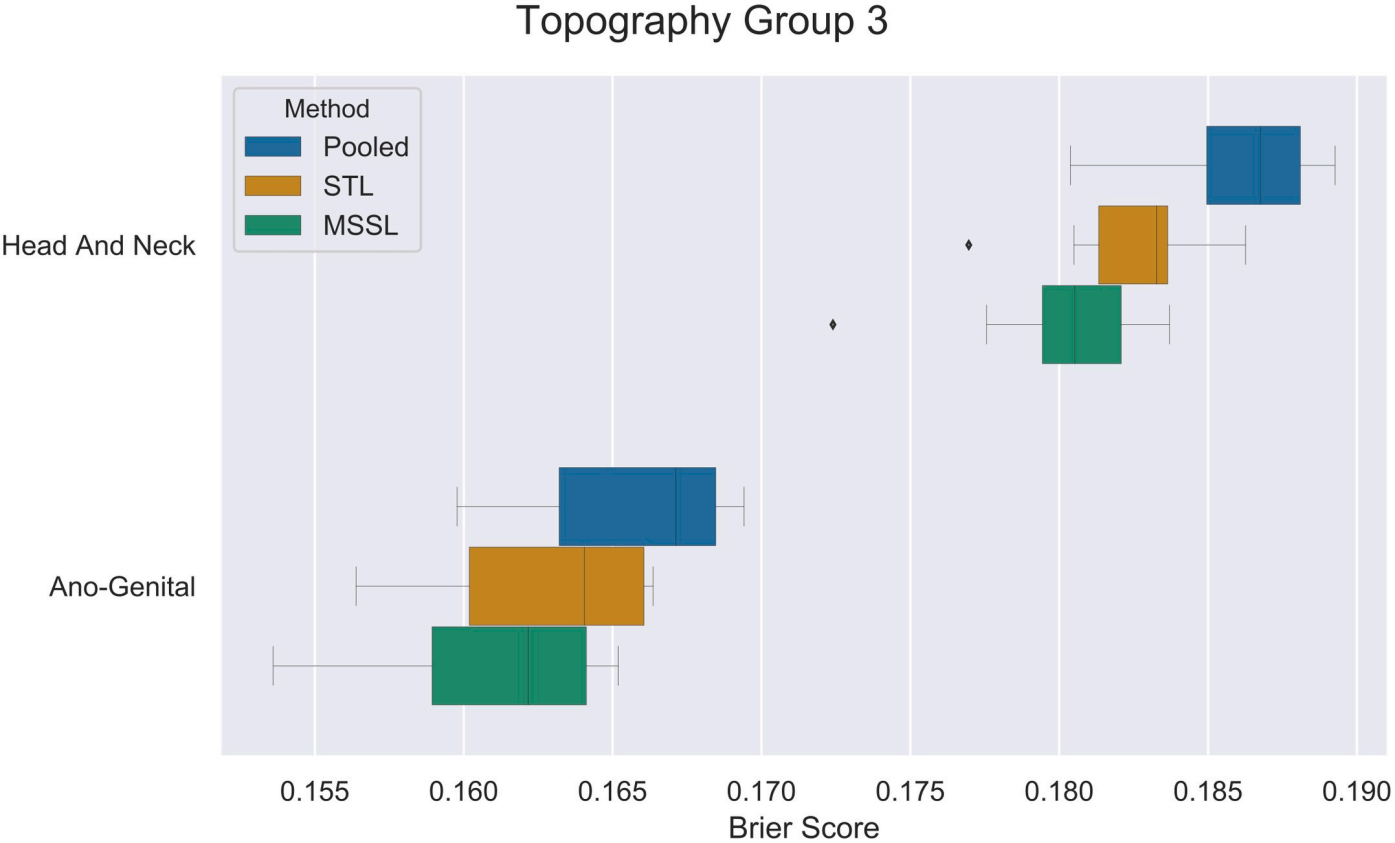
Brier scores (x-axis) for pooled, STL, and MSSL classifiers for *Topography group 3* data split. Boxplots show results from 30 independent runs.

Figs 2, 3 and 4 present per-task performance for the three topography groups. A prevalent pattern across all three topography groups can be observed (particularly for TP2 and TP3): MSSL obtained the best performance (lowest Brier score), followed by STL and then the pooled classifier. For *other/unspecified parts of tongue*, the pooled method presented better performance than MSSL and STL. It indicates that information from other related cancers is helpful for predicting survival for patients diagnosed with *other/unspecified parts of tongue* cancer. Further investigation is required to properly determine the reason for MSSL’s poor performance in *other/unspecified parts of tongue*, in which a significant difference in Brier score was observed. On the other hand, for *oropharynx* and *vagina* (Fig 2), pooled performed much worse than its counterparts, indicating that these two groups do not share much with the other cancer types.

When comparing the three different strategies for task definition based on topography groups (Table 2) in Fig 1, we observe that the definition based on *Topography group 3* shows the best results (lowest Brier score) for all methods. However, even in the coarsest scenario, MSSL produced better results than STL and pooled methods, indicating that intelligent information sharing helps improve 5-year survival predictability at any level of cancer grouping.

### Relative variable importance

**Fig 5:**
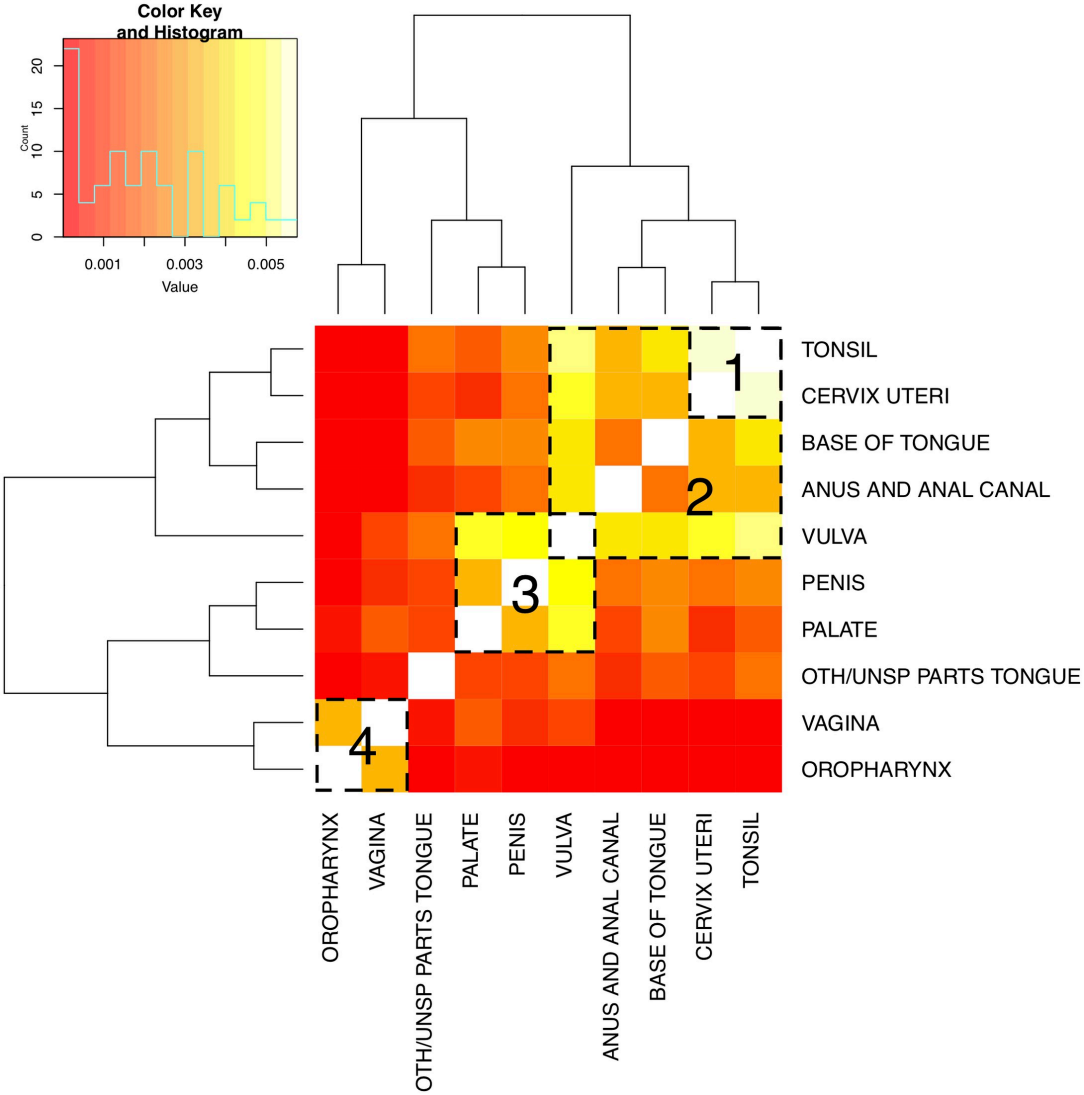
Relative variable importance for STL, MSSL, and Pooled models for the *Topography group 1* experiment. AGE_DX, DAJCCT, DAJCCN, and DAJCCM are the most relevant variables in the Pooled and MSSL models. For the STL model, the importance of the variables is more dependent on the anatomical site, as each model is trained separately.

Fig 5 shows the relative importance of the variables for STL, MSSL, and pooled models considering the task division by *Topography group 1*. The values showed are the average variable relevance computed over 30 independent model runs. To conserve space, the figures for *Topography group 2* and *Topography group 3* are provided in the Appendix A of S1 Appendix. The relative importance of the *i*-th variable (*r*_*i*_) is computed as:

$$\begin{array}{c} r_{i}=\frac{|w_{i}\left|-\text{min}(|w|\right)}{\text{max}(|w|)-\text{min}(|w|)} \end{array}$$ (5)

where **w** is the array of coefficients estimated by the model for all variables, and |*w*_*i*_| denotes the absolute value of the model coefficient associated with the *i*-th variable in the model. For the variables encoded with the one-hot-encoding strategy, |*w*_*i*_| is calculated as the Euclidean norm of the coefficient vector associated with their binary variables. Note that relative importance is 0 for variables not relevant for predicting 5-year survival, and is close to 1 for variables which are highly predictive of the outcome. For MSSL and STL, this metric is computed independently for each task, implying that *r*_*i*_ = 1 in two different tasks does not imply identical importance in each task. The value is relative to the task. Note that we have ignored the sign of the variable while computing importance, as the large majority of the variables used in the model are categorical and not ordinal.

Fig 5 shows the heat map of the relative variable importance for *Topography group 1*. We observe that the age at the time of diagnosis (AGE_DX) is the most relevant variable for Pooled and MSSL models, followed by DAJCCT, DAJCCN, and DAJCCM. These are stage-related variables that indicate the level of severity of the cancer, which clearly reflects in the 5-year survival prediction. We also noticed that for MSSL, registry ID (REG) is relevant for some tasks, indicating that there might be differences across the registries for those anatomical sites. For the STL model, the variables’ importance is more dependent on the anatomical site, which can be explained by the fact that STL approach fits one logistic regression model for each task separately. Therefore, STL model is more susceptible to spurious correlations in the task’s dataset.

To conserve space, relative variable importance heat maps for *Topography group 2* (S1 Fig) and *Topography group 3* (S2 Fig) are presented in Appendix A of S1 Appendix.

(a) STL relative coefficients (variable) importance.

(b) MSSL relative coefficients (variable) importance.

(c) Pooled relative coefficients (variable) importance.

### Learned tasks relationship

**Fig 6:**
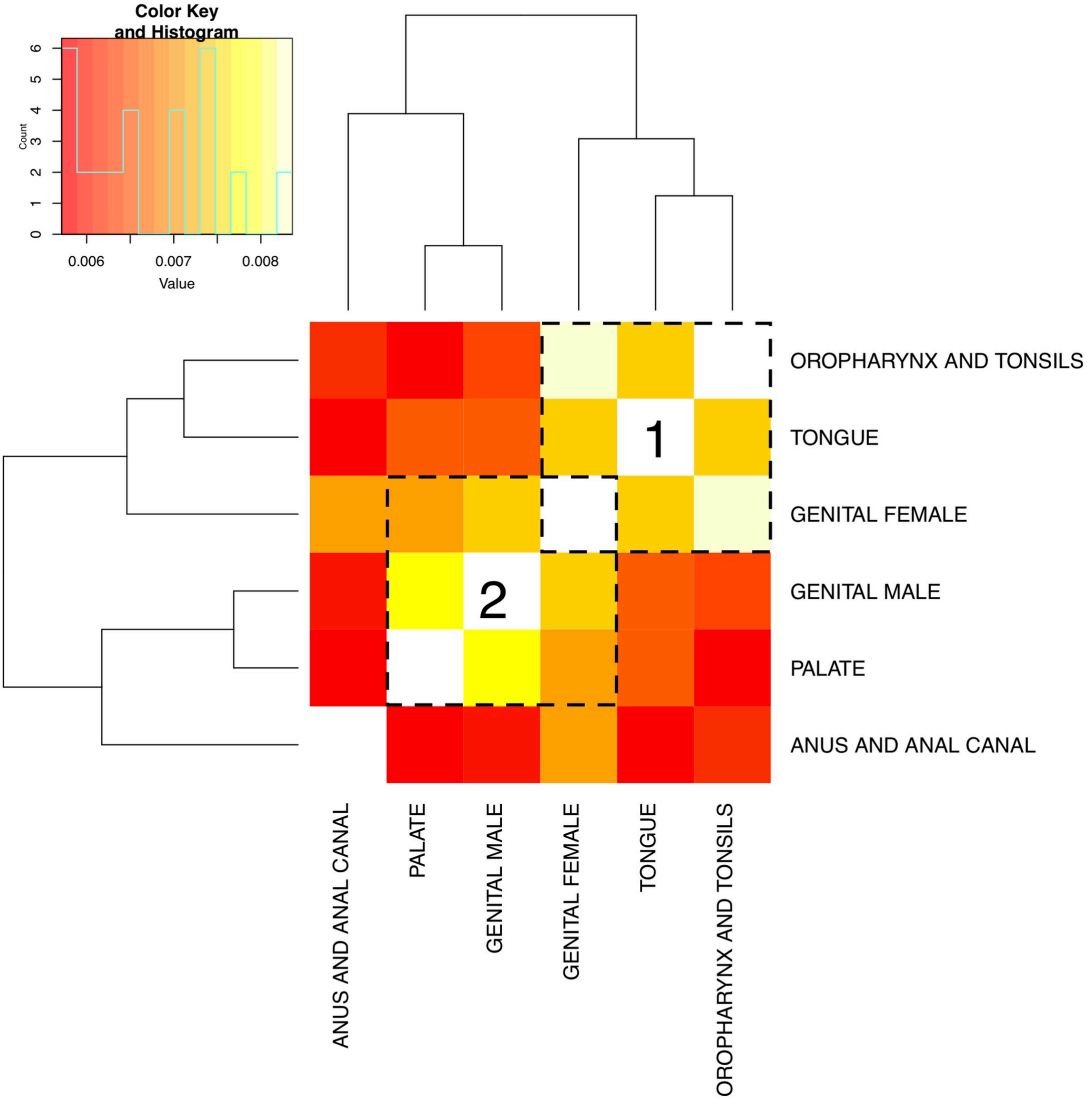
Task relationship learned by MSSL for task split *Topography group 1*. Lighter values indicate stronger relationships. Four groups of cancer sites were found: 1) *tonsil* and *cervix uteri*; 2) *tonsil*, *cervix uteri*, *base of tongue*, *anus and anal canal*, and *vulva*; 3) *vulva*, *penis*, and *palate*; and 4) *vagina* and *oropharynx*. Groups are highlighted in the plot.

**Fig 7:**
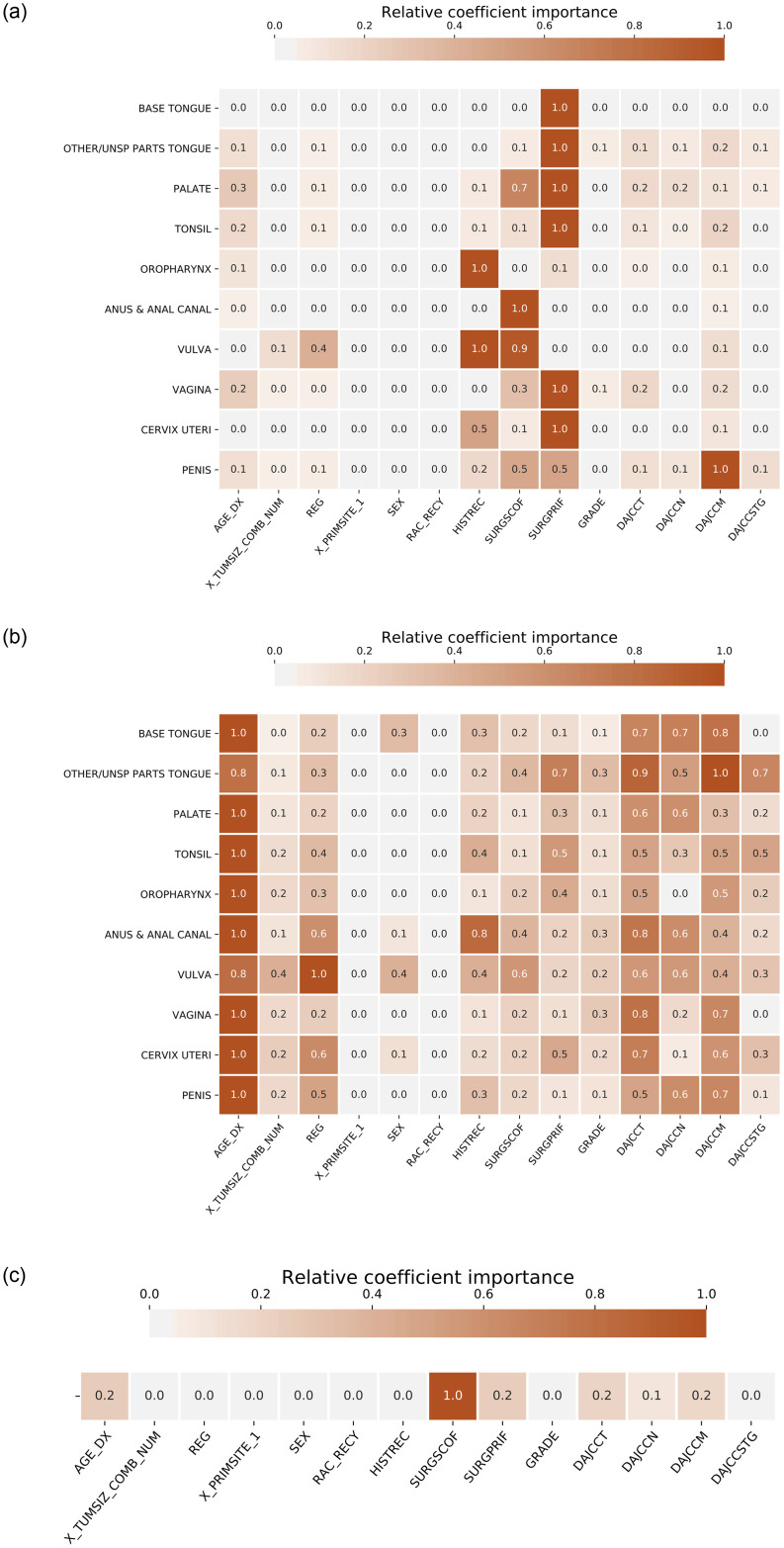
Task relationship learned by MSSL for task *Topography group 2*. Lighter values indicate stronger relationships. *Genital male* and *genital female* are strongly related. *Nasopharynx and palate*, *tongue*, *oropharynx and tonsils* forms a mutually related group. *Oropharynx and tonsils* is related with the majority of tasks, except for *anus and anal canal*. Groups are highlighted in the plot.

Aside from estimating the regression coefficients (**w**), MSSL also learns the precision matrix (**Ω**) that reveals tasks relationship. Precision matrix is the inverse of the covariance matrix, which is estimated based on the regression coefficients as we can see from Eq 4. Therefore, looking at either the precision or the covariance matrix inferred by MSSL can provide relevant information about cancer commonalities, conditioned on the set of variables used in the model. Figs 6 and 7 present the covariance matrices for *Topography group 1* and *Topography group 2* task split. Lighter colors (yellow) are associated with higher tasks commonalities, while darker (red) means weaker relationship between the pair of tasks.

For the *Topography group 1*, MSSL found four groups of related tasks with distinct sizes and magnitudes: 1) *tonsil* and *cervix uteri*; 2) *tonsil*, *cervix uteri*, *base of tongue*, *anus and anal canal*, and *vulva*; 3) *vulva*, *penis*, and *palate*; and 4) *vagina* and *oropharynx*. Firstly, we notice that *tonsil* and *cervix uteri* forms a group (group 1) within a larger group of tasks (group 2). This structure encourages MSSL to share more information between the two tasks in group 1 and with a lesser extent with tasks in the larger group 2. We observe that *vulva* is associated with two different groups of tasks (groups 2 and 3). Therefore, the part of the model associated with *vulva* cancers will share information and also be influenced by data from the two groups of cancers simultaneously. That is possible to be captured in MSSL due to the pairwise nature of the precision matrix learned by the model.

The four groups in Fig 6 appear to be closely related to the 5-year survival rate. For group 1, the survival rate is the highest varying from 68.7 to 72.0%. Group 4 has the lowest survival and varies between 41.9% and 42.4%, while groups 2 and 3 have 5-year survival varying from 62.6%-72.0% and 56.2%-63.5%, respectively. Since we are modeling the 5-year survival prediction, it makes sense that the coefficients of the tasks with similar survival time (tasks in the same group) are more similar.

In the case of *Topography group 2*, Fig 7, *oropharynx and tonsils*, *tongue*, and *genital female* forms a group of mutually related tasks (group 1). A second group of tasks is formed with *genital female*, *genital male*, and *palate* (group 2). Similar to what is observed in Fig 6 with *vulva*, *genital female* appears in two different groups simultaneously. *Anus and anal canal* that appeared in the larger group 2 for *Topography group 1* in Fig 6, now is less related to other cancer groups. This indicates that task definition has a significant effect on how data is shared within the MTL model.

As observed for *Topography group 1*, the two groups are closely related to the 5-year survival rates. The rate for group 1 is the highest, varying from 62.5% to 66.8%, while group 2 has lower survival varying from 56.0% to 58.2%.

### Sample size vs MTL performance improvement

In this study, we have five cancer sites with relative few cases: *other/unsp tongue* (C02), *palate* (C05), *oropharynx* (C10), *vagina* (C52) and *penis* (C60) which all have less then 1000 cases. Five others have relative more cases: *base of tongue* (C01), *tonsil* (C09), *anus & anal canal* (C21), *vulva* (C51), and *cervix uteri* (C53) which have between 2,733 and 9,729 cases, see Table 1. For all cancer sites with relative many cases, the Brier scores are rather similar. While for cancer diagnosis with few cases, MSSL performs clearly better, except for C02 where the pooled model is best, and the STL is doing the worst (see Fig 2).

**Fig 8:**
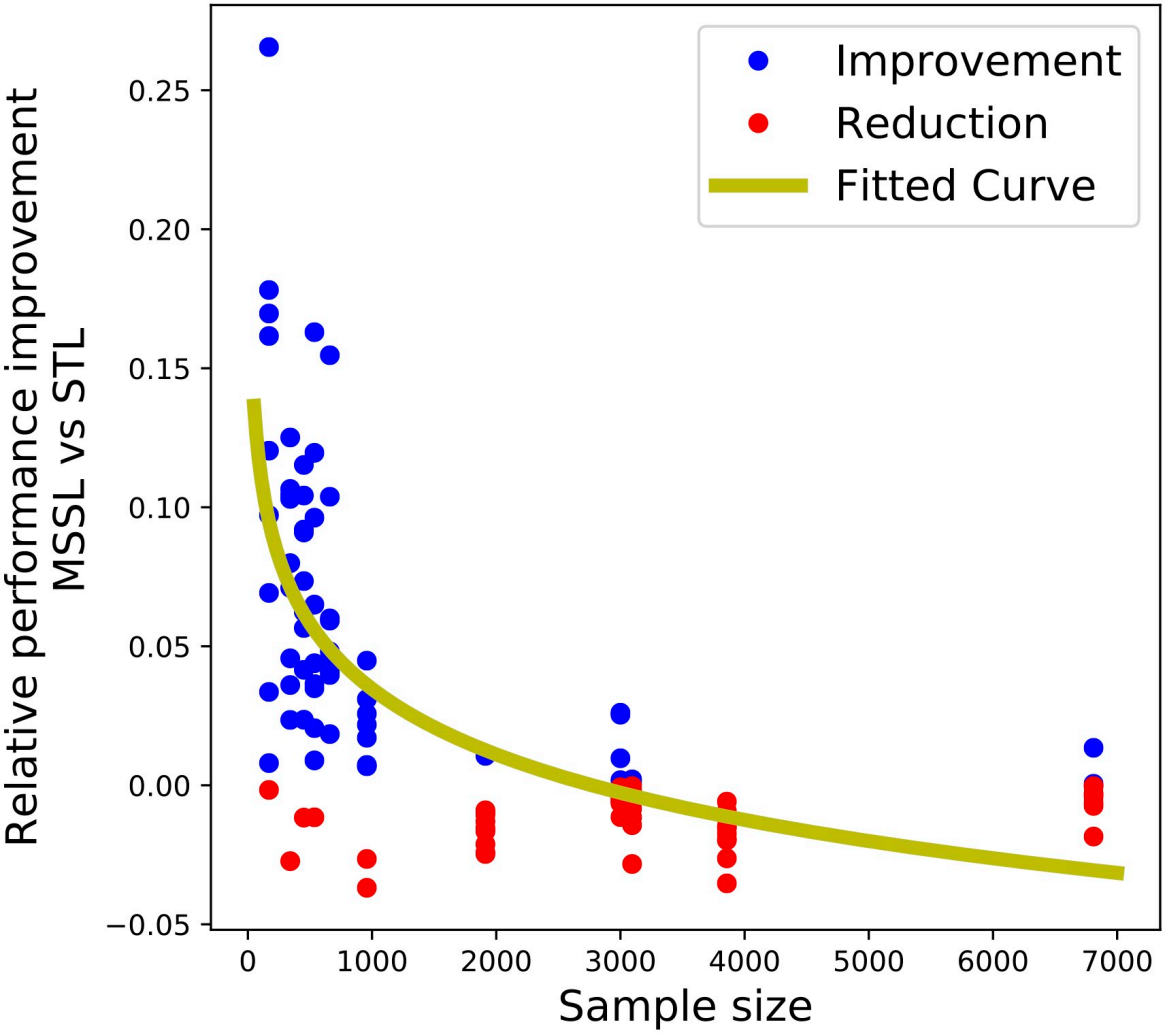
Correlation between task training sample size and MSSL relative performance improvement over STL. Tasks split by *Topography group 1*. MSSL shows higher improvement over STL particularly for tasks with smaller sample sizes.

Fig 8 shows the relationship between the number of training samples in the tasks split by *Topography group 1* and the relative performance improvement (RPI) of MSSL over STL. The performance improvement for task *k* (RPI_k_) is computed as the relative gain in performance of MSSL over STL in terms of the Brier Score:

$$\begin{array}{c} RPI_{\text{k}}=\frac{BS\left(STL_{k}\right)-BS\left(MTL_{k}\right)}{BS(STL_{k})} \end{array}$$ (6)

where BS(⋅) is the Brier score performance on the test set obtained by the method. We clearly observe that small-sample-size tasks benefit the most from the MSSL model. And, as the sample size increase, the difference in performance between MSSL and STL reduces.

## Discussion

In this paper we demonstrated that multitask classifiers achieved significant improvements in predicting survival for the majority of scenarios investigated, when compared to baseline approaches such as single task and pooled classifiers. To the best of our knowledge this is the first demonstration that sharing information across anatomically distinct cancer types can lead to improved predictive survival models.

The present study demonstrates the benefits of leveraging a multitask learning approach to combine clinical data from disparate cancer types in order to improve prediction of cancer patient survival. Previous work has applied MTL approaches to cancer data; however, for the most part, they focused on very specific and homogeneous cancer types, with MTL being deployed for related datasets of the same cancer type. The one exception is the study by [50] in which transfer learning (a machine learning technique related to MTL) was applied across breast and ovarian cancer DNA copy number datasets. While molecular data such as DNA copy number and genetic markers carry relatively high predictive power (in comparison to the type of clinical data used here), they are not ubiquitously deployed in clinical practice for a number of reasons, such as cost or lack of studies demonstrating their translation to courses of action. In contrast, clinical surveillance data, such as the one used in this study and made available by SEER, is ubiquitous in clinical practice. This means that from the machine learning point of view, larger datasets are available; but more importantly, methods trained on this type of data have greater likelihood of actually being deployed to clinical practice to provide decision support to physicians.

Survival and time-to-event data are susceptible to incomplete observations or censoring. While predicting patient survival times based on patient features is of interest, it is important to be able to adapt the learning algorithms to handle censored cases to avoid potential bias [32]. We thus extended IPCW to be used in MTL.

For almost every cancer site selected in this study a common etiological factor linked to infection with human papillomavirus has been reported [51]. Worldwide these virus related cancer sites show an increasing trend, which has been linked to increased exposure to sexually transmitted HPV in the population due to changes in sexual behaviour [52]. Furthermore, some studies indicate a favorable survival pattern in HPV-positive tumors of anal [53], oropharyngeal [54], vaginal [55], and penile cancers [56]. This suggests that these seemingly disparate cancer sites share commonalities which can be leveraged to improve the accuracy of predictive algorithms. To evaluate our hypothesis, we compared the accuracy of a MTL classifier against two baselines. The first baseline consisted of several classifiers (STL classifiers), one for each cancer group. The second baseline consisted of a single classifier applied to the entire cohort (pooled classifier), where the cancer group information used to define the MTL tasks and the different STL classifiers was incorporated as an extra predictive feature. These two baselines represent the approaches most commonly used in research and clinical practice, and both make incorrect assumptions about the data.

In the first baseline (STL), different cancers types were treated individually as if they were independent from each other. The accuracy of the predictions, however, depends heavily on the size of the population, and the independence assumption becomes increasingly more consequential with a decreasing number of patients. Generally, the small sample sizes can lead to severe over-fitting, even for relatively simple models. The one-size-fits-all pooled baseline makes the strong assumption that data from all cancers groups are identically distributed. In other words, this approach completely ignores any differences between individual cancer groups. These pooled models tend to approximate the mean distribution, which will be heavily impacted by the categories with the most samples. MTL approaches make more nuanced assumptions about the relationships between cancer types, allowing them to be treated as non-identically distributed, but also not as entirely independent of one another. In this way certain parameters’ sub-spaces can be shared across all or a subset of cancer groups, whereas others are specific to individual cancer types.

Overall, MTL methods tend to outperform STL methods when there is latent information shared across tasks. Task sample size also plays an important role in model performance. For tasks with large sample sizes, the improvements of MTL over STL can be limited, whereas substantial improvements can be seen for tasks with a smaller number of observations. This, of course, is all dependent on the assumption that there is in fact similarities across the different tasks. If tasks are truly independent, ideally the MTL approach will perform similarly to its STL counterpart.

When tasks are defined at *Topography Group 2 and 3* (see Table 2), MTL improved predictive performance over STL and pooled for the large majority of tasks, meaning that MTL was able to exploit the commonality existing in the tasks (which we speculate is probably due to HPV). While the MTL improvements were seen for all three anatomical cancer groups, significant improvements were not seen in all individual tasks. In our view, these results provide an optimistic perspective on our proposed approach of combining data from disparate cancer types, but there remains many possible directions for improvement.

Analogously to the standard STL approach, the proposed MTL model estimates a set of coefficients (one for each data attribute) for each task, that is, cancer site. The only and crucial distinction is that, during training, the MTL model encourages coefficients of different but related tasks to be similar. However, the model still permits attributes to have very different coefficients for tasks that are not related. Recall that MSSL learns a matrix **Ω** to capture tasks relationship from the data. For example, the importance of “Tumor size” or “Stage” for *base of tongue* could be different to *palate*, if the data say so. The key point of MSSL is that even the model encouraging coefficients of tasks to be close to each other in the parameter space, it is still flexible enough to accommodate any possibly unrelated tasks or particular attributes. Thus, even if “Stage” does not have exactly the same meaning for one particular cancer type compared to the others, including this feature into the MSSL model can still contribute to the improvement of survival prediction.

Multitask learning methods tend to outperform single task learning approaches in the low sample size regime. In such regimes, the implicit information transfer procedure in MSSL diminish the impact of data scarcity, while in large sample sizes, STL models have already enough data to construct an accurate model. In our experiment, we see that the MSSL and STL models have similar performance with regard to Brier score when dealing with larger sample sizes. For cancer sites with less than 1,000 cases, we see marked improvement when using the MSSL methodology.

We attribute the better performance of both MTL and STL methods over pooled methods to the heterogeneity of cancer cases when pooling all data into a single classifier. Even though HPV is a common trigger of the cancer types considered in our cohort, the dissimilarities among the cancer types also play an important role when determining the classifier to use. Thus, the MTL model appears as a suitable candidate to find the correct balance between the one-size-fits-all and completely independent models.

While our working example focuses on HPV-related cancers, there exist numerous other examples in the literature of shared commonalities across distinct cancer types. In this work we explicitly formulated the problem to deal with cancers that share a latent potential causal factor. Many other such examples could similarly be formulated. For example, mutations in the oncogenic signaling protein Ras are found in upwards of 30% of all human cancers [57]. Alternatively we may apply these methods to larger sets of data where we do not explicitly filter the data on known latent causes, but instead extend the MTL framework to help discover the latent connections between cancers automatically. Another possible extension is to use observed survival times instead of binary survival outcomes.

One possible direction is to augment the patients’ information used as predictive features by the machine learning model. Prescribed medications, type and length of treatments, medical notes (unstructured text), medical test results, including images and physiological measurements, are additional sources of relevant information for predicting patient survival. Dealing with unstructured data, e.g., images and text, poses additional challenges, as computational representations of such data need to be extracted. Fortunately, image and text data processing have seen significant advancements in the last decade, particularly due to the development of deep learning models [58]. Going forward, we will investigate how much these additional sources of data can improve the performance of the machine learning models for survival prediction.

In conclusion, we have proposed a new approach for predicting 5-year cancer survival in which data from anatomically distinct cancers can be combined via multitask learning to improve overall prediction accuracy. While this work represents a proof-of-concept demonstration, and extrapolation to larger and broader cohorts remains to be demonstrated, there are a number of potential research directions that could amplify the improvements in performance obtained by MTL. If indeed this type of improvement can be shown to extrapolate across different cohorts, and the improvements can be increased, it has the potential for real-world impact in clinical research and practice.

## Supporting information

S1 File (DOCX)

S1 Appendix (PDF)
