# Supplementary material for: Improving five-year survival prediction via multitask learning across HPV-related cancers
Source: PLoS One. 2020 Nov 16;15(11):e0241225. doi: 10.1371/journal.pone.0241225 (PMC7668590; doi:10.1371/journal.pone.0241225)
Supplement: S1 File — (DOCX) [file pone.0241225.s001.docx]

# Appendix B – Data selection and descriptive analysis.

Data was obtained from the Surveillance, Epidemiology, and End Results (SEER) program*; Nov 2018 Submission;* [*SEER Incidence Data, 1975-2016*](https://seer.cancer.gov/data/)*.*

*We selected data from cancer cases diagnosed from 2004 through 2015 from the following nine SEER registries: Atlanta, Connecticut, Detroit, Hawaii, Iowa, New Mexico, San Francisco-Oakland, Seattle-Puget Sound, and Utah. The following anatomical sites were selected based on the International Classification of Diseases for Oncology, 3rd edition (ICD-O-3) topography: base of tongue (C01.x), other unspecified parts of the tongue (C02*), Palate (C05**), tonsil (C09.x), oropharynx (C10.x), anus and anal canal (C21.x), vulva (C51.x), vagina (C52.x), cervix (C53.x), penis (C60.x). That provided 59,698 cancer cases.*

*We made the following selection*

*
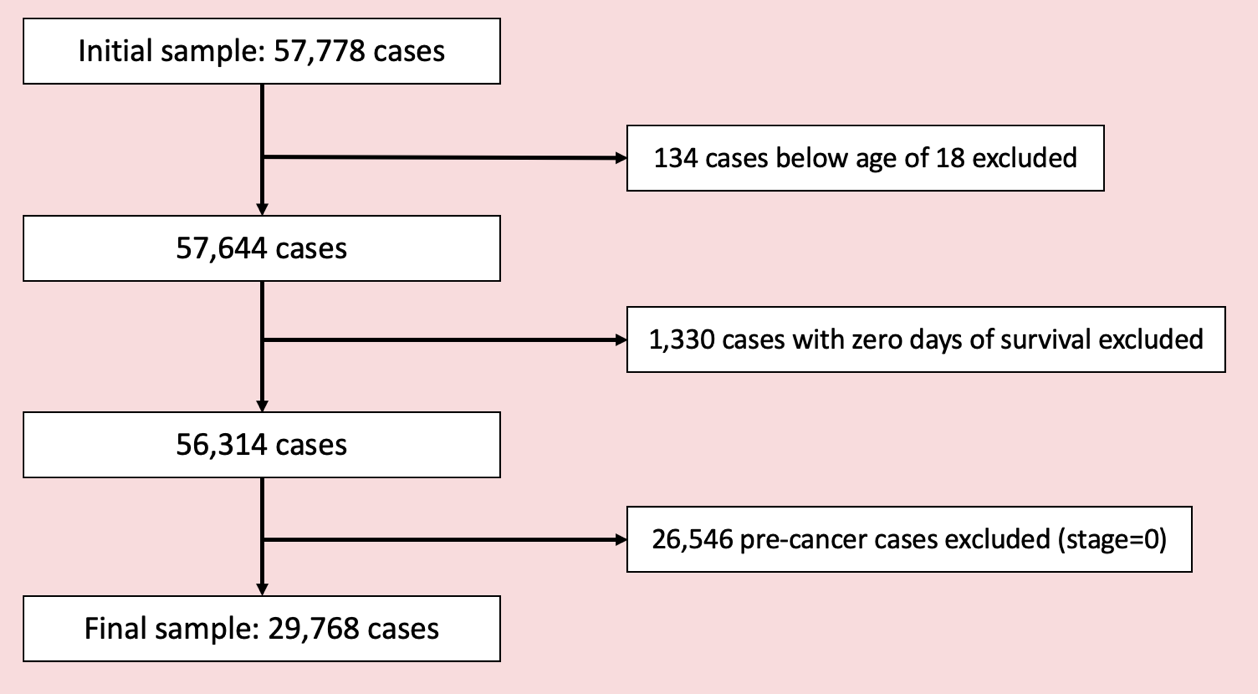
*

** Subsite C02.0-3/9 excluded.*

*** Subsite C05.8/9 excluded.*

The following features were used:

| AGE_DX | Age at diagnosis |
| --- | --- |
| X_PRIMSITE_1 | Primary site (ICD-O-3) |
| DAJCCM | Derived AJCC M 6^th^ ed (2004-2015) |
| DAJCCN | Derived AJCC N 6^th^ ed (2004-2015) |
| DAJCCT | Derived AJCC T 6^th^ ed (2004-2015) |
| DAJCCSTG | Derived AJCC Stage Group 6^th^ ed (2004-2015) |
| GRADE | Grading and differentiation (ICD-O-2) |
| HISTREC | Histology recode – Broad grouping based on histologic type ICD-O-3 |
| RACE1V | Race/ethnicity |
| SEX | Sex of the patient at diagnosis |
| SURGSCOF | The procedure of removal, biopsy, or aspiration of **regional** lymph nodes performed during the initial work-up or first course of therapy at all facilities. |
| X_SURGPRIF_G | surgical procedure that removes and/or destroys tissue of the primary site performed as part of the initial work-up or first course of therapy. |
| REG | SEER Registry |

## **Feature: Age**

|  |  | N | Age | | | Survival (percentage alive) | | |
| --- | --- | --- | --- | --- | --- | --- | --- | --- |
|  |  |  | mean | median | min, max | 1 year | 3 year | 5 years |
| C01 | Base of tongue | 4 421 | 61.0 | 60 | 19, 102 | 85.9 | 69.4 | 63.3 |
| C02 | Other parts of the tongue | 244 | 59.8 | 59 | 26,96 | 85.7 | 67.6 | 59.7 |
| C05 | Palate | 488 | 61.1 | 61 | 20, 101 | 81.7 | 64.0 | 56.2 |
| C09 | Tonsil | 5 511 | 57.9 | 57 | 19, 102 | 89.9 | 77.8 | 72.0 |
| C10 | Oropharynx | 943 | 60.8 | 59 | 31, 97 | 70.6 | 50.8 | 42.2 |
| C21 | Anus and Anal cancal | 4 287 | 60.3 | 59 | 19, 105 | 87.2 | 70.2 | 63.5 |
| C51 | Vulva | 2 733 | 66.1 | 66 | 19, 102 | 85.7 | 70.5 | 62.6 |
| C52 | Vagina | 645 | 65.4 | 64 | 23, 100 | 74.1 | 49.7 | 41.9 |
| C53 | Cervix Uteri | 9 729 | 48.9 | 47 | 19, 103 | 88.1 | 73.9 | 68.7 |
| C60 | Penis | 767 | 65.8 | 66 | 26, 98 | 83.8 | 67.2 | 58.0 |
|  | Total | 29 768 | 57,10 | 57 | 19-105 | 86.7 | 71.5 | 65.2 |

**Kaplan-Meier survival estimates by cancer types**

## **Feature AJCCM (Derived AJCC M, 6th ed (2004-2015))**

Feature’s original coding and distribution Feature’s recoding and distribution

| Code | Description |  | Recode | N | % |  |
| --- | --- | --- | --- | --- | --- | --- |
| 0 | M0 |  | no metastasis | 25 726 | 86.4 |  |
| 10 | M1 |  | metastasis | 1 963 | 6.6 |  |
| 11 | M1a |  | Missing | 2 079 | 7.0 |  |
| 12 | M1b |  |  | 29 768 | 100.0 |  |
| 13 | M1c |  |  |  |  |  |
| 19 | M1 NOS |  |  |  |  |  |
| 88 | Not applicable |  |  |  |  |  |
| 99 | MX |  |  |  |  |  |
|  |  |  |  |  |  |  |

|  |  | | N | | No metastasis | | Metastasis | | Missing | | Survival | | | | | | | | | | | | | | | | | |
| --- | --- | --- | --- | --- | --- | --- | --- | --- | --- | --- | --- | --- | --- | --- | --- | --- | --- | --- | --- | --- | --- | --- | --- | --- | --- | --- | --- | --- |
|  |  | |  |  |  |  |  |  |  |  | No metastasis | | | | | | metastasis | | | | | | Missing | | | | | |
|  |  | |  |  |  |  |  |  |  |  | 1 year | | 3 years | | 5 years | | 1 year | | 3 years | | 5 years | | 1 year | | 3 years | | 5 years | |
| C01 | Base of tongue | 4 421 | | 4 003 | | 171 | | 247 | | 87.6 | | 71.4 | | 65.2 | | 52.6 | | 24.1 | | 18.4 | | 82.0 | | 70.2 | | 64.3 | |  |
| C02 | Other parts of the tongue | 244 | | 221 | | 7 | | 16 | | 87.1 | | 67.4 | | 59.2 | | 66.7 | | 66.7 | | 66.7 | | 74.2 | | 66.8 | | 57.9 | |  |
| C05 | Palate | 488 | | 415 | | 21 | | 52 | | 84.1 | | 65.9 | | 58.0 | | 36.8 | | 14.7 | | 14.7 | | 80.2 | | 67.2 | | 57.4 | |  |
| C09 | Tonsil | 5 511 | | 4 701 | | 159 | | 651 | | 91.2 | | 79.1 | | 73.3 | | 55.5 | | 27.9 | | 22.1 | | 89.1 | | 79.6 | | 73.3 | |  |
| C10 | Oropharynx | 943 | | 804 | | 54 | | 85 | | 74.1 | | 53.7 | | 44.2 | | 25.7 | | 10.1 | | 7.6 | | 67.3 | | 52.7 | | 48.8 | |  |
| C21 | Anus and Anal cancal | 4 287 | | 3 733 | | 311 | | 243 | | 90.4 | | 75.1 | | 68.4 | | 57.3 | | 26.7 | | 20.1 | | 75.7 | | 53.0 | | 46.0 | |  |
| C51 | Vulva | 2 733 | | 2 471 | | 92 | | 170 | | 88.1 | | 72.8 | | 64.6 | | 38.6 | | 19.7 | | 11.4 | | 76.6 | | 62.9 | | 57.3 | |  |
| C52 | Vagina | 645 | | 439 | | 71 | | 135 | | 83.1 | | 61.1 | | 51.8 | | 47.1 | | 15.7 | | 12.6 | | 59.4 | | 30.3 | | 25.0 | |  |
| C53 | Cervix Uteri | 9 729 | | 8 259 | | 1 048 | | 422 | | 93.3 | | 80.9 | | 75.8 | | 53.6 | | 23.9 | | 17.1 | | 72.3 | | 59.4 | | 52.7 | |  |
| C60 | Penis | 767 | | 680 | | 27 | | 60 | | 86.2 | | 69.7 | | 59.8 | | 23.1 | | 4.6 | | 0.0 | | 84.1 | | 69.7 | | 67.3 | |  |

**Kaplan-Meier survival estimates for DAJCCM**

## **Feature AJCCN (Derived AJCC N, 6th ed (2004-2015))**

Feature’s original coding and distribution Feature’s recoding and distribution

| Description |  |  | Description | Recode | N | % |
| --- | --- | --- | --- | --- | --- | --- |
| N0 | no node involment |  | no node involment | 0 | 14 665 | 49.3 |
| N0(i-) | no node involment |  | node involment | 1 | 12 705 | 42.7 |
| N0(i+) | no node involment |  | unknown | 2 | 2 398 | 8.1 |
| N0(mol-) | no node involment |  |  |  | 29 768 | 100.0 |
| N0(mol+) | no node involment |  |  |  |  |  |
| N1 | node involment |  |  |  |  |  |
| N1 NOS | node involment |  |  |  |  |  |
| N1a | node involment |  |  |  |  |  |
| N1b | node involment |  |  |  |  |  |
| N1c | node involment |  |  |  |  |  |
| N1mi | node involment |  |  |  |  |  |
| N2 | node involment |  |  |  |  |  |
| N2 NOS | node involment |  |  |  |  |  |
| N2a | node involment |  |  |  |  |  |
| N2b | node involment |  |  |  |  |  |
| N2c | node involment |  |  |  |  |  |
| N3 | node involment |  |  |  |  |  |
| N3 NOS | node involment |  |  |  |  |  |
| N3a | node involment |  |  |  |  |  |
| N3b | node involment |  |  |  |  |  |
| N3c | node involment |  |  |  |  |  |
| Not applicable | unknown |  |  |  |  |  |
| NX | unknown |  |  |  |  |  |
|  |  |  |  |  |  |  |

|  |  | N | Nodes not involved | Nodes involved | Missing | Survival | | | | | | | | |
| --- | --- | --- | --- | --- | --- | --- | --- | --- | --- | --- | --- | --- | --- | --- |
|  |  |  |  |  |  | Nodes not involved | | | Nodes involved | | | Missing | | |
|  |  |  |  |  |  | 1 y | 3 y | 5 y | 1 y | 3 y | 5 y | 1 y | 3 y | 5 y |
| C01 | Base of tongue | 4 114 | 519 | 3 431 | 164 | 87.7 | 71.6 | 64.9 | 85.8 | 69.1 | 62.8 | 85.8 | 69.1 | 62.8 |
| C02 | Other parts of the tongue | 3 349 | 2 076 | 1 242 | 31 | 92.2 | 74.5 | 67.3 | 81.5 | 62.2 | 53.6 | 81.5 | 62.2 | 53.6 |
| C05 | Palate | 902 | 583 | 245 | 74 | 91.3 | 78.5 | 71.5 | 66.4 | 41.5 | 33.5 | 66.4 | 41.5 | 33.5 |
| C09 | Tonsil | 5 151 | 682 | 3 943 | 526 | 88.7 | 78.1 | 71.1 | 90.2 | 77.2 | 71.8 | 90.2 | 77.2 | 71.8 |
| C10 | Oropharynx | 846 | 168 | 637 | 41 | 74.7 | 53.9 | 43.3 | 70.1 | 50.4 | 41.7 | 70.1 | 50.4 | 41.7 |
| C21 | Anus and Anal cancal | 3 360 | 2 150 | 1 153 | 57 | 91.2 | 78.3 | 71.4 | 81.4 | 57.2 | 49.9 | 81.4 | 57.2 | 49.9 |
| C51 | Vulva | 2 177 | 1 628 | 531 | 18 | 91.9 | 79.8 | 71.3 | 65.9 | 38.1 | 31.1 | 65.9 | 38.1 | 31.1 |
| C52 | Vagina | 542 | 393 | 115 | 34 | 81.3 | 59.5 | 52.2 | 70.6 | 38.9 | 26.8 | 70.6 | 38.9 | 26.8 |
| C53 | Cervix Uteri | 9 549 | 7 168 | 2 123 | 268 | 93.5 | 82.5 | 78.1 | 77.0 | 52.1 | 43.9 | 77.0 | 52.1 | 43.9 |
| C60 | Penis | 698 | 550 | 142 | 6 | 88.8 | 73.2 | 62.4 | 63.4 | 41.6 | 34.8 | 63.4 | 41.6 | 34.8 |

**Kaplan-Meier survival estimates for DAJCCN**

## **Feature AJCCT (Derived AJCC T, 6th ed (2004-2015))**

Feature’s original coding and distribution Feature’s recoding and distribution

|  | DAJCCT |  | Description | Recode | N | % |
| --- | --- | --- | --- | --- | --- | --- |
| T0 | 0 |  | T1 | 1 | 9 987 | 33.6 |
| T1 | 10 |  | T2 | 2 | 8 229 | 27,6 |
| T1a | 12 |  | T3 | 3 | 3 478 | 11,7 |
| T1a1 | 13 |  | T4 | 4 | 2 669 | 9,0 |
| T1a2 | 14 |  | Unknown | 5 | 5 405 | 18,2 |
| T1b | 15 |  |  | Total | 29 768 | 100,0 |
| T1b1 | 16 |  |  |  |  |  |
| T1b2 | 17 |  |  |  |  |  |
| T1 NOS | 19 |  |  |  |  |  |
| T2 | 20 |  |  |  |  |  |
| T2a | 21 |  |  |  |  |  |
| T2b | 22 |  |  |  |  |  |
| T2 NOS | 29 |  |  |  |  |  |
| T3 | 30 |  |  |  |  |  |
| T3a | 31 |  |  |  |  |  |
| T3b | 32 |  |  |  |  |  |
| T3 NOS | 39 |  |  |  |  |  |
| T4 | 40 |  |  |  |  |  |
| T4a | 41 |  |  |  |  |  |
| T4b | 42 |  |  |  |  |  |
| T4 NOS | 49 |  |  |  |  |  |
| T1a NOS | 80 |  |  |  |  |  |
| T1b NOS | 81 |  |  |  |  |  |
| Not applicable | 88 |  |  |  |  |  |
| TX | 99 |  |  |  |  |  |
|  | ~~Total~~ |  |  |  |  |  |

|  |  |  |  |  |  |  |  | Survival (%) | |  | |  |  |  |  |  |  |  |  |  |  |  |  |
| --- | --- | --- | --- | --- | --- | --- | --- | --- | --- | --- | --- | --- | --- | --- | --- | --- | --- | --- | --- | --- | --- | --- | --- |
|  |  | N | T1 | T2 | T3 | T4 | Missing | Stage 1 | | | | Stage 2 | | | Stage 3 | | | Stage 4 | | | Unknown | |  |
|  |  |  |  |  |  |  |  | 1y | 3y | | 5y | 1y | 3y | 5y | 1y | 3y | 5y | 1y | 3y | 5y | 1y | 3y | 5y |
| C01 | Base of tongue | 4 421 | 919 | 1 429 | 476 | 676 | 921 | 94.3 | 83.3 | | 78.0 | 89.3 | 73.4 | 67.1 | 79.6 | 59.8 | 52.5 | 73.1 | 48.0 | 39.7 | 84.9 | 70.5 | 65.2 |
| C02 | Other parts of the tongue | 244 | 80 | 57 | 23 | 42 | 42 | 93.3 | 78.4 | | 69.8 | 87.2 | 69.9 | 60.8 | 72.7 | 55.9 | 48.0 | 71.8 | 41.2 | 37.5 | 90.2 | 73.8 | 63.8 |
| C05 | Palate | 488 | 159 | 114 | 29 | 67 | 119 | 94.1 | 79.6 | | 77.6 | 85.3 | 63.2 | 50.7 | 84.3 | 51.7 | 34.5 | 53.6 | 36.1 | 26.8 | 76.6 | 61.8 | 53.4 |
| C09 | Tonsil | 5 511 | 1 154 | 1 800 | 483 | 761 | 1 313 | 96.2 | 88.4 | | 85.2 | 93.8 | 84.0 | 77.8 | 89.0 | 73.6 | 68.0 | 74.0 | 53.0 | 46.4 | 88.9 | 76.5 | 69.9 |
| C10 | Oropharynx | 943 | 117 | 177 | 88 | 304 | 257 | 85.8 | 69.5 | | 59.2 | 84.4 | 62.3 | 53.1 | 68.4 | 48.0 | 38.9 | 52.9 | 29.1 | 22.6 | 75.8 | 62.1 | 52.8 |
| C21 | Anus and Anal canal | 4 287 | 884 | 1 433 | 533 | 325 | 1 112 | 94.8 | 85.2 | | 79.3 | 90.9 | 75.1 | 66.9 | 81.6 | 55.1 | 47.4 | 74.7 | 47.5 | 40.4 | 82.7 | 66.0 | 60.4 |
| C51 | Vulva | 2 733 | 872 | 864 | 335 | 118 | 544 | 95.6 | 85.9 | | 79.9 | 83.0 | 65.5 | 55.2 | 71.0 | 47.3 | 36.5 | 63.6 | 27.1 | 19.3 | 87.8 | 76.7 | 71.0 |
| C52 | Vagina | 645 | 189 | 182 | 64 | 60 | 150 | 84.0 | 71.7 | | 62.3 | 82.5 | 54.2 | 45.3 | 74.6 | 38.8 | 31.0 | 57.5 | 29.8 | 20.9 | 57.8 | 28.9 | 24.8 |
| C53 | Cervix Uteri | 9 729 | 5 208 | 1 990 | 1 338 | 295 | 898 | 97.1 | 90.7 | | 87.6 | 88.1 | 65.6 | 56.1 | 67.2 | 39.1 | 31.0 | 55.3 | 20.6 | 16.6 | 78.7 | 66.3 | 61.5 |
| C60 | Penis | 767 | 405 | 183 | 109 | 21 | 49 | 90.4 | 75.2 | | 67.0 | 82.3 | 67.4 | 51.4 | 70.6 | 46.9 | 40.7 | 46.3 | 33.1 | 33.1 | 80.7 | 66.0 | 66.0 |

**Kaplan-Meier survival estimates for DAJCCT**

## **Feature AJCCSTG (Derived AJCC STAGE, 6th ed (2004-2015))**

Feature’s original coding Feature’s recoding and distribution

| Description | Code |  | Description | Recode | N | % |
| --- | --- | --- | --- | --- | --- | --- |
| Stage I | 10 |  | Stage I | 1 | 7 702 | 25.9 |
| Stage I NOS | 11 |  | Stage II | 2 | 4 290 | 14.4 |
| Stage IA | 12 |  | Stage III | 3 | 5 240 | 17.6 |
| Stage IA1 | 13 |  | Stage IV | 4 | 9 283 | 31.2 |
| Stage IA2 | 14 |  | Unkown | 5 | 3 253 | 10.9 |
| Stage IB | 15 |  |  |  | 29 768 | 100.0 |
| Stage IB1 | 16 |  |  |  |  |  |
| Stage IB2 | 17 |  |  |  |  |  |
| Stage IC | 18 |  |  |  |  |  |
| Stage IS | 19 |  |  |  |  |  |
| Stage II | 30 |  |  |  |  |  |
| Stage II NOS | 31 |  |  |  |  |  |
| Stage IIA | 32 |  |  |  |  |  |
| Stage IIB | 33 |  |  |  |  |  |
| Stage IIC | 34 |  |  |  |  |  |
| Stage III | 50 |  |  |  |  |  |
| Stage III NOS | 51 |  |  |  |  |  |
| Stage IIIA | 52 |  |  |  |  |  |
| Stage IIIB | 53 |  |  |  |  |  |
| Stage IIIC | 54 |  |  |  |  |  |
| Stage IV | 70 |  |  |  |  |  |
| Stage IV NOS | 71 |  |  |  |  |  |
| Stage IVA | 72 |  |  |  |  |  |
| Stage IVB | 73 |  |  |  |  |  |
| Stage IVC | 74 |  |  |  |  |  |
| Not applicable | 88 |  |  |  |  |  |
| Stage Occult | 90 |  |  |  |  |  |
| Stage Unknown | 99 |  |  |  |  |  |

|  |  |  |  |  |  |  |  | Survival (Percentage alive) | | | | | | | | | | | | | | |
| --- | --- | --- | --- | --- | --- | --- | --- | --- | --- | --- | --- | --- | --- | --- | --- | --- | --- | --- | --- | --- | --- | --- |
|  |  | N | Stage  I | Stage II | Stage III | Stage IV | Missing | Stage I | | | Stage II | | | Stage III | | | Stage IV | | | Unknown | | |
|  |  |  |  |  |  |  |  | 1y | 3y | 5y | 1y | 3y | 5y | 1y | 3y | 5y | 1y | 3y | 5y | 1y | 3y | 5y |
| C01 | Base of tongue | 4 421 | 211 | 258 | 589 | 3 004 | 359 | 93.5 | 79.3 | 72.9 | 88.1 | 75.0 | 67.9 | 89.5 | 71.6 | 65.6 | 84.3 | 67.6 | 61.1 | 86.8 | 72.0 | 67.6 |
| C02 | Other parts of the tongue | 244 | 44 | 27 | 29 | 129 | 15 | 92.7 | 75.6 | 68.5 | 96.2 | 79.4 | 79.4 | 88.7 | 69.0 | 38.3 | 79.7 | 62.0 | 54.2 | 93.1 | 62.1 | 62.1 |
| C05 | Palate | 488 | 129 | 63 | 44 | 163 | 89 | 96.0 | 85.6 | 83.0 | 91.6 | 69.7 | 61.3 | 95.1 | 67.5 | 50.1 | 61.4 | 40.8 | 33.7 | 84.8 | 69.7 | 58.7 |
| C09 | Tonsil | 5 511 | 358 | 501 | 805 | 3 407 | 440 | 96.2 | 88.6 | 84.5 | 93.1 | 86.8 | 77.7 | 94.9 | 83.7 | 77.7 | 87.9 | 74.2 | 69.2 | 88.0 | 74.4 | 66.1 |
| C10 | Oropharynx | 943 | 45 | 65 | 88 | 633 | 112 | 88.4 | 69.0 | 61.2 | 87.0 | 70.2 | 63.3 | 80.0 | 61.4 | 47.8 | 65.6 | 44.8 | 36.6 | 74.8 | 59.0 | 50.8 |
| C21 | Anus and Anal cancal | 4 287 | 752 | 1 197 | 1 063 | 314 | 961 | 95.2 | 87.6 | 82.5 | 91.5 | 76.9 | 68.3 | 86.1 | 63.6 | 56.6 | 57.1 | 26.4 | 19.9 | 86.6 | 70.4 | 64.5 |
| C51 | Vulva | 2 733 | 814 | 621 | 508 | 231 | 559 | 96.5 | 89.0 | 82.6 | 88.8 | 73.3 | 61.7 | 78.5 | 50.3 | 40.8 | 49.8 | 25.5 | 20.3 | 87.6 | 76.4 | 70.3 |
| C52 | Vagina | 645 | 159 | 146 | 95 | 110 | 135 | 88.9 | 76.0 | 70.0 | 84.5 | 59.1 | 51.0 | 78.9 | 49.1 | 33.6 | 54.8 | 24.8 | 18.0 | 57.5 | 28.1 | 23.0 |
| C53 | Cervix Uteri | 9 729 | 4 835 | 1 258 | 1 899 | 1 225 | 512 | 98.4 | 93.5 | 90.8 | 92.1 | 72.5 | 63.9 | 84.3 | 60.9 | 51.8 | 55.2 | 24.6 | 18.5 | 74.4 | 59.2 | 51.7 |
| C60 | Penis | 767 | 355 | 154 | 120 | 67 | 71 | 92.6 | 78.1 | 68.7 | 88.0 | 72.5 | 56.5 | 75.3 | 52.2 | 46.3 | 42.4 | 19.5 | 15.6 | 83.5 | 72.7 | 70.5 |

**Kaplan-Meier survival estimates for DAJCCSTG**

## **Feature Grade**

**Feature’s original coding Feature’s recoding and distribution**

| Description | |  |  |  | Code |  | Description | Recode | N | % |
| --- | --- | --- | --- | --- | --- | --- | --- | --- | --- | --- |
| 1,00 | Grade I; well differentiated; | |  |  | 1 |  | Grade I | 1 | 2 711 | 9.1 |
| 2,00 | Grade II moderately differentiated | | |  | 2 |  | Grade II | 2 | 9 172 | 30.8 |
| 3,00 | Grade III; poorly differentiated | |  |  | 3 |  | Grade III | 3 | 8 364 | 28.1 |
| 4,00 | Grade IV; undifferentiated; anaplastic | | |  | 4 |  | Grade IV | 4 | 412 | 1.4 |
| 5,00 | T-cell; T-precursor |  |  |  | 5 |  | Missing | 5 | 9 109 | 30.6 |
| 6,00 | B-cell; Pre-B; B-Precursor | |  |  | 6 |  |  |  | 29 768 | 100.0 |
| 7,00 | Null cell; Non T-non B; | |  |  | 7 |  |  |  |  |  |
| 8,00 | N K cell (natural killer cell) | |  |  | 8 |  |  |  |  |  |
| 9,00 | cell type not determined, not stated or not applicable | | | | 9 |  |  |  |  |  |

|  |  |  | Grade  I | Grade  II | Grade III | Grade IV | Unknown | Survival | | | | | | | | | | | | | | |
| --- | --- | --- | --- | --- | --- | --- | --- | --- | --- | --- | --- | --- | --- | --- | --- | --- | --- | --- | --- | --- | --- | --- |
|  |  | N |  |  |  |  |  | Grade I | | | Grade II | | | Grade III | | | Grade IV | | | Grade IV | | |
|  |  |  |  |  |  |  |  | 1y | 3y | 5y | 1y | 3y | 5y | 1y | 3y | 5y | 1y | 3y | 5y | 1y | 3y | 5y |
| C01 | Base of tongue | 4 421 | 165 | 1 188 | 1 565 | 39 | 1 464 | 0.85 | 0.65 | 0.60 | 0.83 | 0.65 | 0.59 | 0.91 | 0.75 | 0.69 | 0.84 | 0.73 | 0.69 | 0.84 | 0.67 | 0.61 |
| C02 | Other parts of the tongue | 244 | 24 | 114 | 46 | 1 | 59 | 0.80 | 0.52 | 0.52 | 0.87 | 0.66 | 0.59 | 0.80 | 0.63 | 0.53 | 1.00 | 1.00 | 1.00 | 0.89 | 0.78 | 0.65 |
| C05 | Palate | 488 | 77 | 199 | 95 | 5 | 112 | 0.87 | 0.79 | 0.73 | 0.86 | 0.65 | 0.57 | 0.73 | 0.49 | 0.36 | 0.80 | 0.57 | 0.57 | 0.78 | 0.65 | 0.60 |
| C09 | Tonsil | 5 511 | 145 | 1 547 | 2 065 | 60 | 1 694 | 0.81 | 0.68 | 0.60 | 0.89 | 0.77 | 0.72 | 0.92 | 0.80 | 0.75 | 0.91 | 0.79 | 0.76 | 0.89 | 0.76 | 0.69 |
| C10 | Oropharynx | 943 | 38 | 273 | 244 | 11 | 377 | 0.64 | 0.52 | 0.43 | 0.65 | 0.40 | 0.31 | 0.72 | 0.54 | 0.45 | 0.62 | 0.62 | 0.44 | 0.75 | 0.57 | 0.49 |
| C21 | Anus and Anal cancal | 4 287 | 422 | 1 415 | 1 105 | 54 | 1 291 | 0.91 | 0.75 | 0.69 | 0.87 | 0.70 | 0.62 | 0.85 | 0.67 | 0.60 | 0.80 | 0.50 | 0.47 | 0.88 | 0.73 | 0.67 |
| C51 | Vulva | 2 733 | 571 | 817 | 332 | 19 | 994 | 0.89 | 0.77 | 0.67 | 0.82 | 0.64 | 0.56 | 0.75 | 0.55 | 0.46 | 0.89 | 0.69 | 0.69 | 0.90 | 0.77 | 0.70 |
| C52 | Vagina | 645 | 48 | 161 | 174 | 24 | 238 | 0.78 | 0.61 | 0.55 | 0.75 | 0.53 | 0.45 | 0.72 | 0.49 | 0.40 | 0.61 | 0.51 | 0.38 | 0.75 | 0.46 | 0.39 |
| C53 | Cervix Uteri | 9 729 | 1 041 | 3 153 | 2 618 | 195 | 2 722 | 0.97 | 0.91 | 0.89 | 0.91 | 0.77 | 0.72 | 0.83 | 0.63 | 0.57 | 0.75 | 0.60 | 0.51 | 0.87 | 0.75 | 0.70 |
| C60 | Penis | 767 | 180 | 305 | 120 | 4 | 158 | 0.88 | 0.72 | 0.68 | 0.84 | 0.65 | 0.52 | 0.71 | 0.54 | 0.44 | 1.00 | 1.00 | 1.00 | 0.89 | 0.76 | 0.68 |

**Kaplan-Meier survival estimates for Grade**

## **Feature Histrec**

**Feature’s original coding Feature’s recoding and distribution**

| Description |  | Code |  | Description | Recode | N | Percent |
| --- | --- | --- | --- | --- | --- | --- | --- |
| 00  8000-8009 | unspecified neoplasms | 0 |  | squamous cell neoplams (SCC) | 1 | 23 891 | 80.3 |
| 01  8010-8049 | epithelial neoplasms, NOS | 1 |  | adenomas and adenocarcinomas ACC) | 2 | 2 914 | 9.8 |
| 02  8050-8089 | squamous cell neoplams | 2 |  | Other | 3 | 2 963 | 10.0 |
| 03  8090-8119 | basal cell neoplams | 3 |  |  |  | 29768 | 100.0 |
| 04  8120-8139 | transitional cell papillomas and carcinomas | 4 |  |  |  |  |  |
| 05  8140-8389 | adenomas and adenocarcinomas | 5 |  |  |  |  |  |
| 06  8390-8429 | adnexal and skin appendage neoplams | 6 |  |  |  |  |  |
| 07  8430-8439 | mucoepidermoid neoplasms | 7 |  |  |  |  |  |
| 08  8440-8499 | cystic, mucinous and serous neoplams | 8 |  |  |  |  |  |
| 09  8500-8549 | ductal and lobular neoplams | 9 |  |  |  |  |  |
| 10  8550-8559 | acinar cell neoplasms | 10 |  |  |  |  |  |
| 11  8560-8579 | complex epithelial neoplams | 11 |  |  |  |  |  |
| 15  8720-8799 | nevi and melanomas | 15 |  |  |  |  |  |
| 21  8930-8999 | complex mixed and stromal neoplasms | 21 |  |  |  |  |  |
| 40 9590-9599 | malignant lymphomas, NOS or diffuse | 40 |  |  |  |  |  |
| 41 9650-9669 | hodgkin lymphomas | 41 |  |  |  |  |  |
| 42 9670-9699 | hodg - mature b-cell lymphomaskin lymphomas | 42 |  |  |  |  |  |
| 43 9700-9719 | nhl - mature t and nk-cell lymphomas | 43 |  |  |  |  |  |
| 44 9720-9729 | nhl - precursor cell lymphoblastic lymphoma | 44 |  |  |  |  |  |
| 45-9730-9749 | plasma cell tumors | 45 |  |  |  |  |  |
| 50 9820-9839 | lymphoid leukemias (C42.1) | 50 |  |  |  |  |  |

|  |  | N | SCC | ACC | Other | Survival (percent alive) | | | | | | | | |
| --- | --- | --- | --- | --- | --- | --- | --- | --- | --- | --- | --- | --- | --- | --- |
|  |  |  |  |  |  | SCC | | | ACC | | | Other | | |
|  |  |  |  |  |  | 1y | 3y | 5y | 1y | 3y | 5y | 1y | 3y | 5y |
| C01 | Base of tongue | 4 421 | 4 184 | 41 | 196 | 86.0 | 69.1 | 63.0 | 84.4 | 66.3 | 62.2 | 84.4 | 78.2 | 69.4 |
| C02 | Other parts of the tongue | 244 | 229 | 1 | 14 | 85.3 | 66.1 | 58.1 | 0.0 | 0.0 | 0.0 | 92.3 | 83.1 | 71.2 |
| C05 | Palate | 488 | 352 | 37 | 99 | 76.4 | 54.2 | 44.1 | 94.4 | 85.1 | 77.8 | 95.8 | 89.9 | 88.5 |
| C09 | Tonsil | 5 511 | 4 951 | 12 | 548 | 89.8 | 77.0 | 71.2 | 75.0 | 66.2 | 66.2 | 91.4 | 84.1 | 77.6 |
| C10 | Oropharynx | 943 | 895 | 3 | 45 | 70.5 | 50.6 | 41.7 | 66.7 | 33.3 | 33.3 | 72.4 | 56.6 | 56.6 |
| C21 | Anus and Anal cancal | 4 287 | 3 519 | 387 | 381 | 88.9 | 73.8 | 67.9 | 78.9 | 50.6 | 40.0 | 79.8 | 59.3 | 49.2 |
| C51 | Vulva | 2 733 | 2 064 | 35 | 634 | 83.6 | 68.1 | 59.9 | 85.1 | 59.7 | 49.8 | 92.3 | 78.2 | 71.2 |
| C52 | Vagina | 645 | 412 | 94 | 139 | 74.3 | 51.5 | 41.9 | 86.7 | 64.0 | 60.8 | 65.1 | 35.0 | 29.1 |
| C53 | Cervix Uteri | 9 729 | 6 559 | 2 303 | 867 | 87.8 | 72.9 | 67.3 | 91.0 | 80.1 | 76.2 | 83.1 | 64.5 | 58.6 |
| C60 | Penis | 767 | 726 | 1 | 40 | 83.0 | 66.2 | 56.8 | 0.0 | 0.0 | 0.0 | 97.4 | 88.1 | 83.8 |

**Kaplan-Meier survival estimates for Histrec**

## **Feature RACE1V**

**Feature’s original coding Feature’s recoding and distribution**

| Description | Code |  | Description | Recode | N | Percentage |
| --- | --- | --- | --- | --- | --- | --- |
| White | 1 |  | White | 1 | 23 927 | 80.4 |
| Black | 2 |  | Black | 2 | 3 381 | 11.4 |
| American Indian, Aleutian, Alaskan Native or Eskimo | 3 |  | Other | 3 | 2 460 | 8.3 |
| Chinese | 4 |  |  |  | 29 768 | 100.0 |
| Japanese | 5 |  |  |  |  |  |
| Filipino | 6 |  |  |  |  |  |
| Hawaiian | 7 |  |  |  |  |  |
| Korean | 8 |  |  |  |  |  |
| Vietnamese | 10 |  |  |  |  |  |
| Laotian | 11 |  |  |  |  |  |
| Hmong | 12 |  |  |  |  |  |
| Kampuchean (including Khmer and Cambodian | 13 |  |  |  |  |  |
| Thai | 14 |  |  |  |  |  |
| Asian Indian or Pakistani, NOS | 15 |  |  |  |  |  |
| Asian Indian | 16 |  |  |  |  |  |
| Pakistani | 17 |  |  |  |  |  |
| Micronesian, NOS | 20 |  |  |  |  |  |
| Chamorran | 21 |  |  |  |  |  |
| Guamanian, NOS | 22 |  |  |  |  |  |
| Polynesian, NOS | 25 |  |  |  |  |  |
| Tahitian |  |  |  |  |  |  |
| Samoan | 27 |  |  |  |  |  |
| Tongan | 28 |  |  |  |  |  |
| Melanesian, NOS |  |  |  |  |  |  |
| Fiji Islander | 31 |  |  |  |  |  |
| New Guinean |  |  |  |  |  |  |
| Other Asian, including Asian, NOS and Oriental, NOS | 96 |  |  |  |  |  |
| Pacific Islander, NOS | 97 |  |  |  |  |  |
| Other | 98 |  |  |  |  |  |
| Unknown | 99 |  |  |  |  |  |

|  |  | N | White | Black | Other | Survival | | | | | | | | |
| --- | --- | --- | --- | --- | --- | --- | --- | --- | --- | --- | --- | --- | --- | --- |
|  |  |  |  |  |  | White | | | Black | | | Other | | |
|  |  |  |  |  |  | 1y | 3y | 5y | 1y | 3y | 5y | 1y | 3y | 5y |
| C01 | Base of tongue | 4 421 | 3 902 | 317 | 202 | 86.8 | 71.2 | 64.9 | 73.1 | 48.5 | 41.7 | 88.8 | 71.7 | 68.0 |
| C02 | Other parts of the tongue | 244 | 205 | 20 | 19 | 86.6 | 69.3 | 61.2 | 63.2 | 21.1 | 14.0 | 100.0 | 93.6 | 85.4 |
| C05 | Palate | 488 | 385 | 75 | 28 | 82.9 | 65.1 | 57.7 | 71.8 | 55.1 | 45.7 | 91.8 | 76.1 | 63.9 |
| C09 | Tonsil | 5 511 | 4 653 | 511 | 347 | 91.2 | 79.9 | 74.0 | 77.7 | 56.5 | 49.4 | 91.2 | 80.1 | 76.8 |
| C10 | Oropharynx | 943 | 737 | 161 | 45 | 73.0 | 54.8 | 45.5 | 59.2 | 35.0 | 27.3 | 72.4 | 50.1 | 50.1 |
| C21 | Anus and Anal cancal | 4 287 | 3 534 | 558 | 195 | 87.1 | 71.3 | 64.7 | 87.1 | 65.5 | 57.8 | 89.1 | 66.9 | 59.4 |
| C51 | Vulva | 2 733 | 2 328 | 237 | 168 | 84.8 | 69.6 | 61.5 | 90.2 | 77.9 | 69.5 | 91.1 | 71.7 | 66.2 |
| C52 | Vagina | 645 | 499 | 83 | 63 | 74.2 | 51.0 | 43.5 | 70.2 | 37.5 | 32.4 | 78.3 | 55.1 | 42.0 |
| C53 | Cervix Uteri | 9 729 | 7 049 | 1 353 | 1 327 | 89.2 | 75.5 | 70.4 | 80.9 | 63.2 | 57.4 | 89.7 | 76.2 | 70.6 |
| C60 | Penis | 767 | 635 | 66 | 66 | 83.6 | 67.0 | 57.1 | 82.5 | 65.3 | 59.8 | 87.3 | 74.0 | 69.1 |

**Kaplan-Meier survival estimates for Race1V**

## **Feature Sex**

Original coding kept

1: Male

2:Female

|  |  | N | Male | Female | Survival | | | | | |
| --- | --- | --- | --- | --- | --- | --- | --- | --- | --- | --- |
|  |  |  |  |  | Male | | | Female | | |
|  |  |  |  |  | 1y | 3y | 5y | 1y | 3y | 5y |
| C01 | Base of tongue | 4 421 | 3 712 | 709 | 0,87 | 0,70 | 0,64 | 0,79 | 0,65 | 0,59 |
| C02 | Other parts of the tongue | 244 | 167 | 77 | 0,85 | 0,66 | 0,56 | 0,88 | 0,70 | 0,66 |
| C05 | Palate | 488 | 300 | 188 | 0,78 | 0,61 | 0,52 | 0,87 | 0,70 | 0,63 |
| C09 | Tonsil | 5 511 | 4 499 | 1 012 | 0,91 | 0,78 | 0,73 | 0,86 | 0,76 | 0,69 |
| C10 | Oropharynx | 943 | 735 | 208 | 0,71 | 0,52 | 0,44 | 0,68 | 0,48 | 0,38 |
| C21 | Anus and Anal cancal | 4 287 | 1 707 | 2 580 | 0,85 | 0,67 | 0,59 | 0,88 | 0,73 | 0,67 |
| C51 | Vulva | 2 733 | 0 | 2 733 | – | – | – | 0,86 | 0,70 | 0,62 |
| C52 | Vagina | 645 | 0 | 645 | – | – | – | 0,74 | 0,50 | 0,42 |
| C53 | Cervix Uteri | 9 729 | 0 | 9 729 | – | – | – | 0,88 | 0,74 | 0,69 |
| C60 | Penis | 767 | 767 | 0 | 0,84 | 0,67 | 0,58 | – | – | – |

**Kaplan-Meier survival estimates for Sex**

## **Feature SURGSCOF**

**Feature’s original coding Feature’s recoding and distribution**

| Description | Code |  | Description | Recode | N | Percent | |  |
| --- | --- | --- | --- | --- | --- | --- | --- | --- |
| No regional lymph nodes removed or aspirated; diagnosed at autopsy. | 0 |  | No regional lymph nodes removed or aspirated; diagnosed at autopsy. | 0 | 18 565 | | 62.4 | |
| Biopsy or aspiration of regional lymph node, NOS | 1 |  | Biopsy or aspiration of regional lymph node, NOS/biopsy Sentinel node | 1 | 2 086 | | 7.0 | |
| Sentinel lymph node biopsy [only] | 2 |  | Removed regional nodes | 2 | 8 820 | | 29.6 | |
| Number of regional lymph nodes removed unknown, not stated; regional lymph nodes removed, NOS | 3 |  | Uknown | 3 | 297 | | 1.0 | |
| 1 to 3 regional lymph nodes removed | 4 |  |  |  | 29 768 | | 100.0 | |
| 4 or more regional lymph nodes removed | 5 |  |  |  |  |  | |  |
| Sentinel node biopsy and code 3, 4, or 5 at same time or timing not noted | 6 |  |  |  |  |  | |  |
| Sentinel node biopsy and code 3, 4, or 5 at different times | 7 |  |  |  |  |  | |  |
| Unknown or not applicable; death certificate only | 9 |  |  |  |  |  | |  |

|  |  | N | No nodes removed | Biopsy | Nodes removed | Unknown | Survival (Percent alive) | | | | | | | | | | | | |
| --- | --- | --- | --- | --- | --- | --- | --- | --- | --- | --- | --- | --- | --- | --- | --- | --- | --- | --- | --- |
|  |  |  |  |  |  |  | No nodes removed | | | Biopsy | | | Nodes removed | | | Unknown | | |  |
|  |  |  |  |  |  |  | 1y | 3y | 5y | 1y | 3y | 5y | 1y | 3y | 5y | 1y | 3y | 5y |  |
| C01 | Base of tongue | 4 421 | 2 386 | 813 | 1 180 | 42 | 81.8 | 62.6 | 56.0 | 86.2 | 71.5 | 66.2 | 93.7 | 81.9 | 75.6 | 92.1 | 79.4 | 79.4 |  |
| C02 | Other parts of the tongue | 244 | 105 | 24 | 112 | 3 | 80.5 | 61.6 | 53.4 | 87.2 | 76.7 | 68.6 | 89.7 | 69.4 | 61.3 | 100.0 | 100.0 | 100.0 |  |
| C05 | Palate | 488 | 401 | 18 | 63 | 6 | 81.4 | 65.5 | 57.5 | 50.0 | 32.4 | 24.3 | 91.5 | 62.6 | 55.1 | 100.0 | 100.0 | 100.0 |  |
| C09 | Tonsil | 5 511 | 2 890 | 728 | 1 754 | 139 | 86.4 | 72.5 | 65.6 | 90.1 | 75.5 | 69.7 | 95.7 | 86.9 | 83.3 | 90.3 | 82.2 | 70.5 |  |
| C10 | Oropharynx | 943 | 591 | 132 | 203 | 17 | 64.5 | 44.2 | 35.1 | 73.8 | 57.1 | 51.5 | 85.5 | 66.1 | 57.4 | 81.3 | 65.8 | 65.8 |  |
| C21 | Anus and Anal cancal | 4 287 | 3 785 | 110 | 378 | 14 | 87.5 | 71.6 | 65.7 | 84.5 | 63.8 | 55.2 | 85.1 | 59.7 | 45.1 | 92.9 | 77.4 | 77.4 |  |
| C51 | Vulva | 2 733 | 1 464 | 172 | 1 084 | 13 | 83.0 | 69.4 | 62.1 | 94.0 | 80.1 | 72.3 | 88.0 | 70.7 | 61.9 | 84.0 | 42.0 | 42.0 |  |
| C52 | Vagina | 645 | 538 | 13 | 86 | 8 | 71.4 | 47.5 | 39.6 | 75.0 | 35.0 | 35.0 | 90.5 | 64.6 | 55.9 | 75.0 | 50.0 | 50.0 |  |
| C53 | Cervix Uteri | 9 729 | 5 828 | 47 | 3 805 | 49 | 82.3 | 64.3 | 58.0 | 81.2 | 48.7 | 40.6 | 97.1 | 88.8 | 85.1 | 77.1 | 62.2 | 43.3 |  |
| C60 | Penis | 767 | 577 | 29 | 155 | 6 | 84.4 | 67.8 | 58.0 | 75.0 | 45.0 | 45.0 | 82.9 | 69.4 | 61.4 | 100.0 | 77.8 | 77.8 |  |

**Kaplan-Meier survival estimates for SURGSCOF**

## **Feature SURGPRIF_GEN**

**Feature’s original coding Feature’s recoding and distribution**

| Description | Code |  | Description | Recode | N | Percent |
| --- | --- | --- | --- | --- | --- | --- |
| None; no surgical procedure of primary site; diagnosed at autopsy only | 0 |  | No surgery | 1 | 14 453 | 48,6 |
| Site-specific codes. Tumor destruction; no pathologic specimen or unknown whether there is a pathologic specimen | 10 |  | Surgery | 2 | 15 229 | 51,2 |
| Site-specific codes. Resection; pathologic specimen | 20 |  | Unknown | 3 | 86 | 0,3 |
| Surgery, NOS. A surgical procedure to the primary site was done, but no information on the type of surgical procedure is provided. | 90 |  |  |  | 29 768 | 100.0 |
| Unknown if surgery performed; death certificate only | 99 |  |  |  |  |  |

|  |  | N | No surgery | Surgery | Unknown | Survival (percent alive) | | | | | |  | |  | |  |
| --- | --- | --- | --- | --- | --- | --- | --- | --- | --- | --- | --- | --- | --- | --- | --- | --- |
|  |  |  |  |  |  | No Surgery | | | Surgery | | | Unknown | | | | |
|  |  |  |  |  |  | 1y | 3y | 5y | 1y | 3y | 5y | 1y | 3y | | 5y | |
| C01 | Base of tongue | 4 421 | 3 396 | 1 017 | 8 | 0,84 | 0,67 | 0,61 | 0,92 | 0,77 | 0,71 | 1,00 | 0,78 | | 0,47 | |
| C02 | Other parts of the tongue | 244 | 92 | 152 | 0 | 0,81 | 0,64 | 0,50 | 0,89 | 0,69 | 0,64 | – | – | | – | |
| C05 | Palate | 488 | 240 | 247 | 1 | 0,70 | 0,50 | 0,41 | 0,93 | 0,77 | 0,71 | 1,00 | 1,00 | | 1,00 | |
| C09 | Tonsil | 5 511 | 2 789 | 2 703 | 19 | 0,85 | 0,69 | 0,62 | 0,95 | 0,87 | 0,82 | 0,71 | 0,53 | | 0,53 | |
| C10 | Oropharynx | 943 | 718 | 219 | 6 | 0,66 | 0,47 | 0,38 | 0,84 | 0,67 | 0,59 | 0,84 | 0,67 | | 0,59 | |
| C21 | Anus and Anal cancal | 4 287 | 2 439 | 1 841 | 7 | 0,85 | 0,67 | 0,60 | 0,90 | 0,75 | 0,67 | 0,85 | 0,85 | | 0,85 | |
| C51 | Vulva | 2 733 | 432 | 2 290 | 11 | 0,61 | 0,39 | 0,32 | 0,90 | 0,76 | 0,68 | 0,81 | 0,31 | | 0,31 | |
| C52 | Vagina | 645 | 418 | 221 | 6 | 0,67 | 0,41 | 0,32 | 0,88 | 0,66 | 0,61 | 0,67 | 0,33 | | 0,33 | |
| C53 | Cervix Uteri | 9 729 | 3 865 | 5 841 | 23 | 0,75 | 0,50 | 0,42 | 0,97 | 0,90 | 0,86 | 0,73 | 0,67 | | 0,67 | |
| C60 | Penis | 767 | 64 | 698 | 5 | 0,64 | 0,54 | 0,49 | 0,86 | 0,69 | 0,59 | 1,00 | 0,71 | | 0,71 | |

**Kaplan-Meier survival estimates for SURGPRIF_GEN**

## **Feature REG**

Original coding kept

| Registry | Code | N | Percent |
| --- | --- | --- | --- |
| San Francisco-Oakland SMSA | 1 501 | 4 322 | 14.5 |
| Connecticut | 1 502 | 4 001 | 13.4 |
| Metropolitan Detroit | 1 520 | 4 428 | 14.9 |
| Hawaii | 1 521 | 1 376 | 4.6 |
| Iowa | 1 522 | 3 478 | 11.7 |
| New Mexico | 1 523 | 2 037 | 6.8 |
| Seattle (Puget Sound) | 1 525 | 5 074 | 17.1 |
| Utah | 1 526 | 1 740 | 5.9 |
| Metropolitan Atlanta | 1 527 | 3 312 | 1.1 |

|  |  | N | San Francisco-Oakland | Connecticut | Met. Detroit | Hawaii | Iowa | Survival (Percent alive) | | | | | | | | | | | | | | |
| --- | --- | --- | --- | --- | --- | --- | --- | --- | --- | --- | --- | --- | --- | --- | --- | --- | --- | --- | --- | --- | --- | --- |
|  |  |  |  |  |  |  |  | San Francisco-Oakland SMSA | | | Connecticut | | | Metropolitan  Detroit | | | Hawaii | | | Iowa | | |
|  |  |  |  |  |  |  |  | 1y | 3y | 5y | 1y | 3y | 5y | 1y | 3y | 5y | 1y | 3y | 5y | 1y | 3y | 5y |
| C01 | Base of tongue | 2 446 | 650 | 562 | 640 | 203 | 391 | 86.7 | 70.2 | 63.6 | 87.0 | 69.6 | 63.4 | 81.6 | 64.3 | 58.6 | 88.5 | 70.4 | 62.3 | 87.3 | 72.0 | 66.1 |
| C02 | Other parts of the tongue | 2 075 | 517 | 481 | 415 | 241 | 421 | 90.9 | 68.9 | 68.9 | 83.8 | 52.3 | 45.3 | 75.0 | 55.4 | 45.4 | 100.0 | 100.0 | 85.7 | 84.6 | 76.2 | 62.3 |
| C05 | Palate | 572 | 147 | 120 | 168 | 39 | 98 | 81.2 | 59.1 | 53.9 | 78.6 | 58.5 | 52.1 | 76.6 | 63.4 | 54.6 | 76.2 | 65.7 | 53.7 | 89.6 | 82.6 | 74.2 |
| C09 | tonsil | 3 120 | 749 | 723 | 742 | 241 | 665 | 90.8 | 79.8 | 73.7 | 89.4 | 77.9 | 74.4 | 85.5 | 69.1 | 62.5 | 88.2 | 75.2 | 69.6 | 92.2 | 78.2 | 71.7 |
| C10 | Oropharynx | 519 | 114 | 122 | 154 | 36 | 93 | 73.6 | 59.2 | 51.3 | 67.9 | 51.0 | 39.6 | 63.8 | 41.3 | 32.1 | 74.7 | 50.3 | 46.4 | 70.9 | 48.0 | 42.8 |
| C21 | Anus and Anal cancal | 1 998 | 587 | 429 | 487 | 126 | 369 | 88.9 | 72.1 | 65.2 | 87.9 | 72.1 | 66.1 | 85.2 | 66.2 | 58.6 | 88.5 | 70.9 | 64.5 | 87.3 | 68.6 | 61.7 |
| C51 | Vulva | 1 408 | 243 | 371 | 359 | 90 | 345 | 83.3 | 68.0 | 60.2 | 81.9 | 64.4 | 58.3 | 85.1 | 70.2 | 60.9 | 90.0 | 71.0 | 64.4 | 82.8 | 68.5 | 60.2 |
| C52 | Vagina | 350 | 92 | 78 | 75 | 26 | 79 | 81.5 | 59.3 | 51.1 | 79.7 | 46.1 | 37.2 | 68.6 | 47.1 | 40.1 | 69.7 | 51.8 | 42.0 | 73.1 | 50.3 | 39.7 |
| C53 | Cervix Uteri | 5 426 | 1 366 | 1 134 | 1 438 | 498 | 990 | 90.0 | 77.7 | 72.2 | 87.5 | 73.9 | 69.2 | 84.1 | 68.2 | 62.5 | 87.3 | 75.2 | 69.5 | 88.4 | 74.3 | 68.3 |
| C60 | Penis | 428 | 99 | 110 | 89 | 23 | 107 | 87.9 | 77.0 | 70.3 | 79.8 | 68.4 | 53.7 | 87.6 | 70.3 | 66.0 | 96.6 | 72.4 | 62.4 | 85.0 | 67.8 | 54.7 |

|  |  | N | New Mexico | Seattle | Utah | Met. Atlanta | Survival | | | | | | | | | | | |
| --- | --- | --- | --- | --- | --- | --- | --- | --- | --- | --- | --- | --- | --- | --- | --- | --- | --- | --- |
|  |  |  |  |  |  |  | New Mexico | | | Seattle | | | Utah | | | Metropolitan Atlanta | | |
|  |  |  |  |  |  |  | 1y | 3y | 5y | 1y | 3y | 5y | 1y | 3y | 5y | 1y | 3y | 5y |
| C01 | Base of tongue | 1 823 | 254,0 | 831,0 | 252,0 | 486,0 | 82.6 | 65.8 | 56.2 | 87.3 | 72.1 | 66.3 | 83.4 | 73.3 | 68.0 | 87.8 | 68.8 | 63.7 |
| C02 | Other parts of the tongue | 97 | 22,0 | 44,0 | 15,0 | 16,0 | 76.2 | 70.6 | 63.5 | 92.7 | 72.4 | 61.3 | 92.3 | 83.1 | 83.1 | 79.3 | 45.9 | 45.9 |
| C05 | Palate | 179 | 30,0 | 71,0 | 35,0 | 43,0 | 80.0 | 50.9 | 42.1 | 85.7 | 62.3 | 58.4 | 84.4 | 76.9 | 57.0 | 85.4 | 63.7 | 57.2 |
| C09 | tonsil | 2 183 | 336,0 | 1 014,0 | 293,0 | 540,0 | 90.3 | 76.6 | 69.9 | 91.4 | 80.9 | 74.5 | 89.9 | 82.5 | 77.1 | 90.7 | 79.6 | 74.2 |
| C10 | Oropharynx | 370 | 73,0 | 141,0 | 46,0 | 110,0 | 75.0 | 63.9 | 46.0 | 74.4 | 52.6 | 46.1 | 75.0 | 55.9 | 40.5 | 69.7 | 48.7 | 45.6 |
| C21 | Anus and Anal cancal | 1 790 | 279,0 | 748,0 | 220,0 | 543,0 | 84.5 | 71.7 | 62.5 | 86.4 | 70.2 | 63.6 | 85.3 | 71.1 | 64.1 | 89.2 | 71.4 | 66.0 |
| C51 | Vulva | 1 000 | 159,0 | 427,0 | 159,0 | 255,0 | 88.0 | 66.4 | 56.8 | 90.1 | 75.5 | 70.8 | 89.3 | 78.9 | 65.3 | 87.9 | 75.9 | 65.5 |
| C52 | Vagina | 232 | 42,0 | 96,0 | 34,0 | 60,0 | 68.3 | 54.9 | 45.2 | 71.0 | 45.7 | 41.8 | 84.6 | 59.4 | 59.4 | 68.7 | 37.7 | 29.9 |
| C53 | Cervix Uteri | 4 193 | 754,0 | 1 614,0 | 627,0 | 1 198,0 | 88.8 | 72.3 | 66.3 | 90.8 | 77.8 | 74.0 | 88.3 | 76.1 | 87.3 | 69.5 | 63.8 | 64.1 |
| C60 | Penis | 296 | 88,0 | 88,0 | 59,0 | 61,0 | 79.9 | 57.9 | 52.3 | 83.6 | 67.2 | 56.0 | 76.2 | 59.2 | 48.1 | 82.8 | 62.9 | 62.9 |

**Kaplan-Meier survival estimates for REG**
