# Supplementary material for: Improving five-year survival prediction via multitask learning across HPV-related cancers
Source: PLoS One. 2020 Nov 16;15(11):e0241225. doi: 10.1371/journal.pone.0241225 (PMC7668590; doi:10.1371/journal.pone.0241225)
Supplement: S1 Appendix — (PDF) [file pone.0241225.s002.pdf]

S1 Appendix

A Relative variable importance

In this section, we show the relative variable importance for all three models for the *Topography group 2* (S1 Fig) and *Topography group 3* (S2 Fig) task splits.

S1 **Fig.** Relative variable importance for STL, MSSL and Pooled models for the *Topography group 2* experiment.

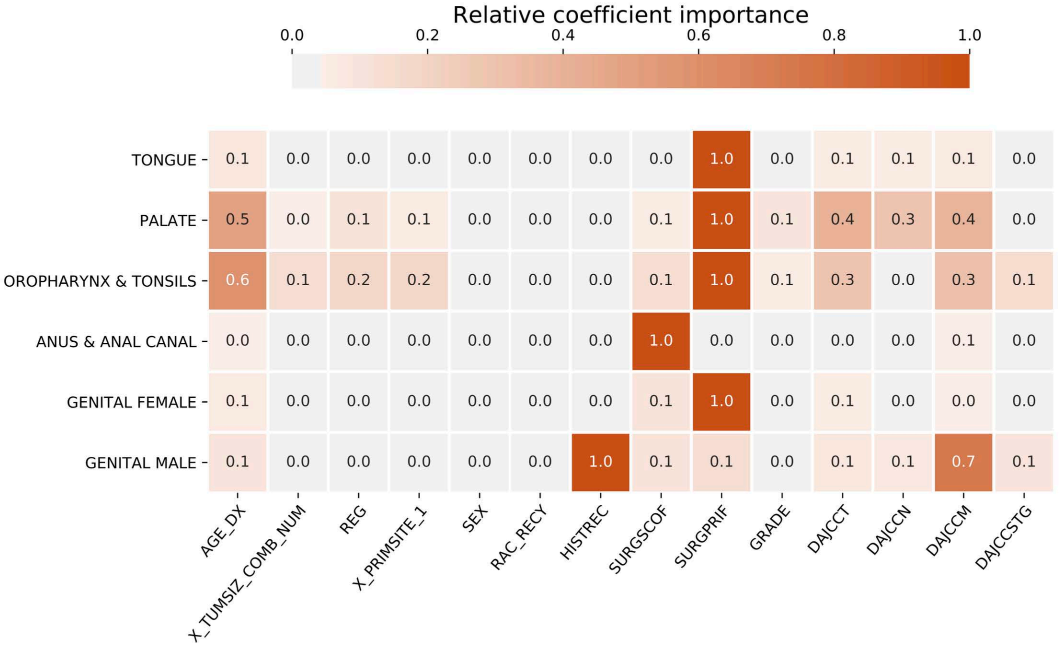

(a) STL relative coefficients (variable) importance.

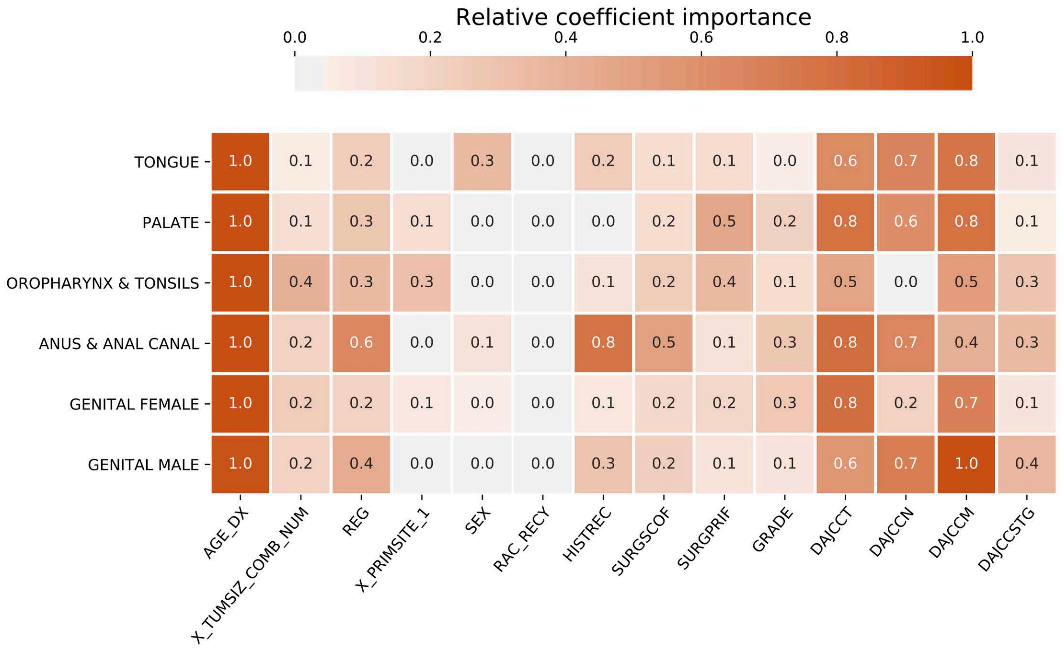

(b) MSSL relative coefficients (variable) importance.

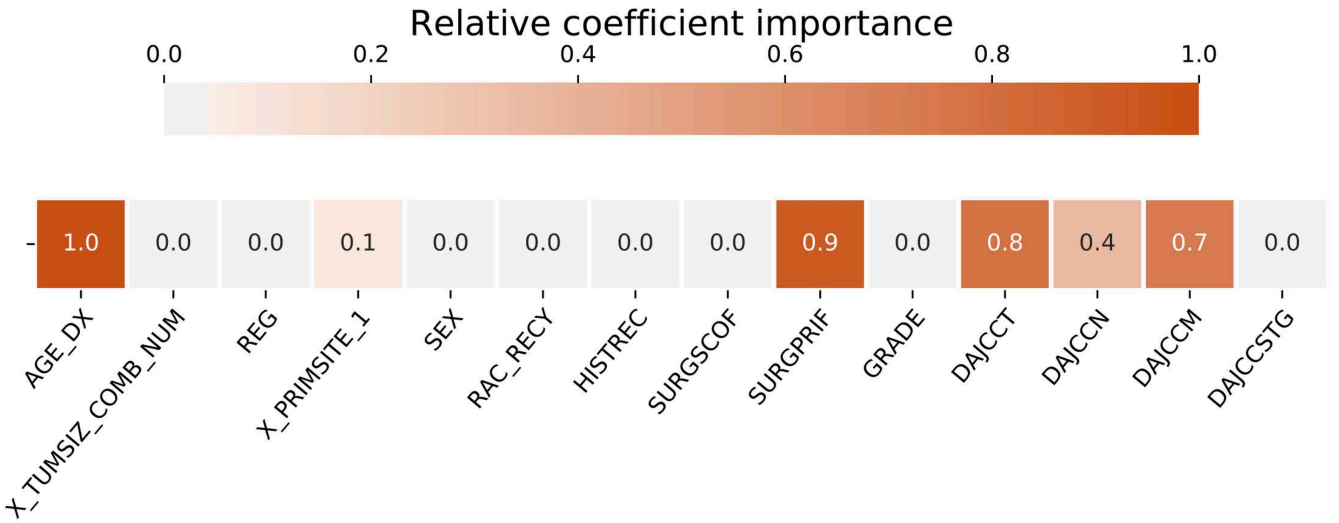

(c) Pooled relative coefficients (variable) importance.

S2 **Fig.** Relative variable importance for STL, MSSL and Pooled models for the *Topography group 3* experiment.

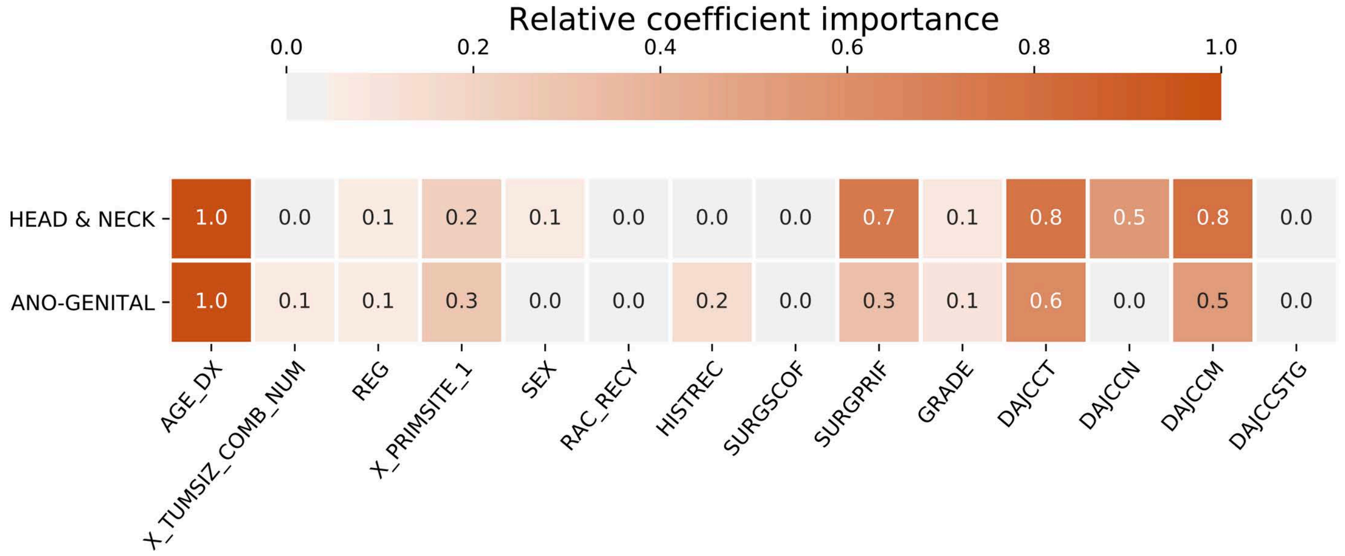

(a) STL relative coefficients (variables) importance.

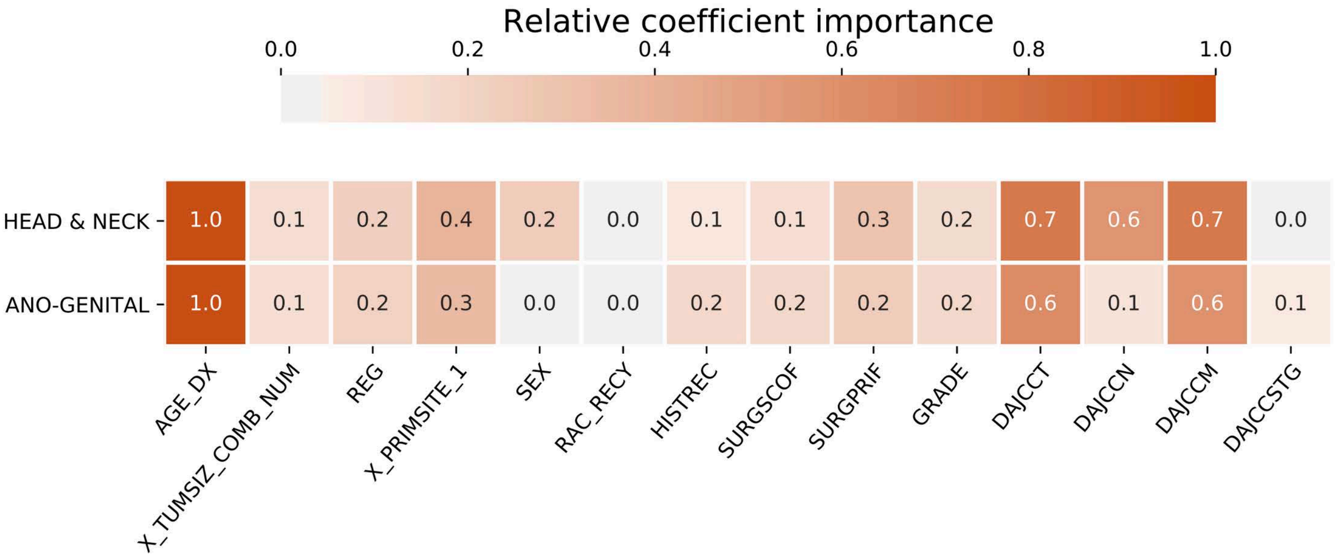

(b) MSSL relative coefficients (variables) importance.

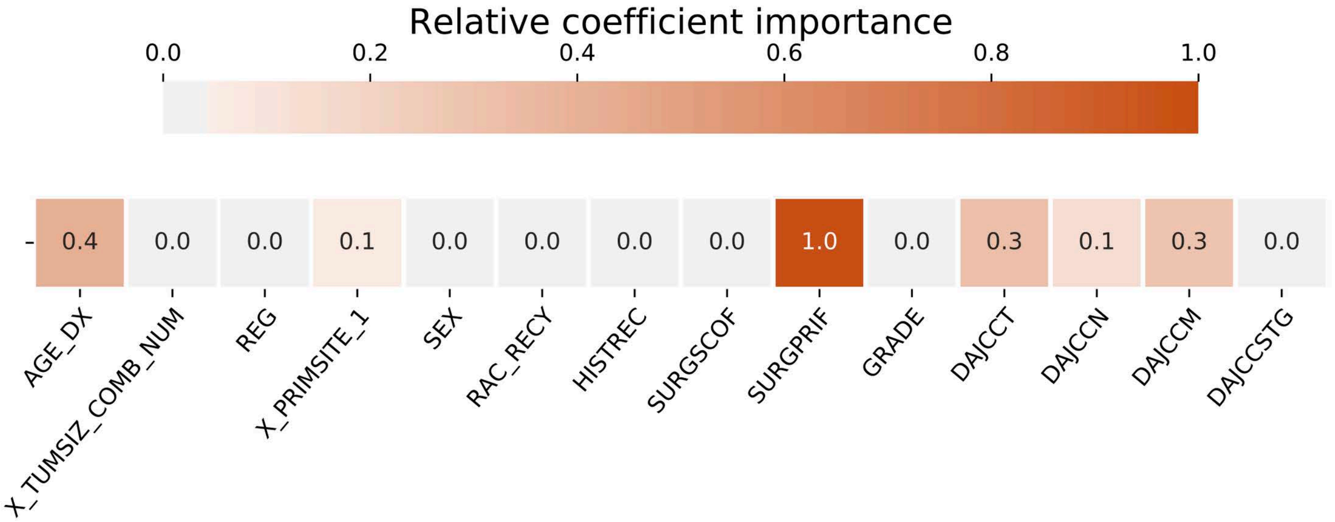

(c) Pooled relative coefficients (variables) importance.

B Effect size statistics

S1 **Table.** Cohen's d and common language effect size (CLES) statistics for TP1 experiment. Large positive values for Cohen's d are indicative of larger effect of the treatment (method 2) over the control (method 1). CLES shows the percentage of the runs in which the treatment (method 2) provided better (lower) Brier score than the control (method 1).

	Cohen's d			CLES		
Method 1 ⟶	Pooled	STL	Pooled	Pooled	STL	Pooled
	vs.	vs.	vs.	vs.	vs.	vs.
Method 2 ⟶	MSSL	MSSL	STL	MSSL	MSSL	STL
Anus And Anal Canal	1.61	0.60	1.44	0.93	0.60	0.90
Base Of Tongue	1.70	-1.58	2.33	0.83	0.20	0.93
Cervix Uteri	6.25	-2.00	6.44	1.00	0.23	1.00
Oropharynx	3.35	1.77	2.29	0.97	0.87	0.87
Other/Unsp. P. Tongue	-1.73	2.99	-3.00	0.20	1.00	0.10
Palate	1.33	1.82	0.21	0.73	1.00	0.70
Penis	0.25	1.95	-0.67	0.67	0.87	0.37
Tonsil	1.03	-2.76	1.12	1.00	0.17	1.00
Vagina	5.22	1.55	4.00	0.97	0.83	0.93
Vulva	3.99	-0.94	3.93	1.00	0.30	0.97

S2 **Table.** Cohen's d and common language effect size (CLES) statistics for TP2 experiment. Large positive values for Cohen's d are indicative of larger effect of the treatment (method 2) over the control (method 1). CLES shows the percentage of the runs in which the treatment (method 2) provided better (lower) Brier score than the control (method 1).

	Cohen's d			CLES		
Method 1 ⟶	Pooled	STL	Pooled	Pooled	STL	Pooled
	vs.	vs.	vs.	vs.	vs.	vs.
Method 2 ⟶	MSSL	MSSL	STL	MSSL	MSSL	STL
Anus And Anal Canal	2.77	1.08	2.10	0.93	0.60	0.87
Genital Female	5.94	0.16	5.96	1.00	0.57	1.00
Genital Male	-0.28	1.71	-1.13	0.47	0.80	0.30
Palate	0.16	0.35	0.04	0.53	0.53	0.47
Oropharynx And Tonsils	4.60	1.20	4.03	1.00	0.70	0.97
Tongue	2.33	0.42	1.82	0.87	0.57	0.83

S3 **Table.** Cohen's d and common language effect size (CLES) statistics for TP3 experiment. Large positive values for Cohen's d are indicative of larger effect of the treatment (method 2) over the control (method 1). CLES shows the percentage of the runs in which the treatment (method 2) provided better (lower) Brier score than the control (method 1).

	Cohen's d			CLES		
Method 1 ⟶	Pooled	STL	Pooled	Pooled	STL	Pooled
	vs.	vs.	vs.	vs.	vs.	vs.
Method 2 ⟶	MSSL	MSSL	STL	MSSL	MSSL	STL
Ano-Genital	11.41	4.73	6.47	1.00	1.00	1.00
Head And Neck	9.19	5.51	8.29	1.00	1.00	1.00
